# The Redox Paradox of Natural Supplements in Cancer: A Narrative Review to Guide Clinical Practice

**DOI:** 10.3390/antiox15070809

**Published:** 2026-06-28

**Authors:** Pierrick Martinez, Enrique A. Martinez Mosqueira, Lionel Gillot, William Makis, Casey Peavler, Antonio Vega-Galvez, Fabrice Joulia, William B. Grant

**Affiliations:** 1 Laboratoire Jeunesse-Activité Physique et Sportive-Santé (J-AP2S), Univ Toulon, 83041 Toulon, France; pierrick-martinez@etud.univ-tln.fr (P.M.); joulia@univ-tln.fr (F.J.); 2 Oasis Santé, Medical Center, 30700 Uzès, France; martinezea@proton.me; 3 Department of Nutritional Research and Development, Nutri-Logics SA, 9911 Troisvierges, Luxembourg; lionel.gillot@nutri-logics.com; 4 We The People Health and Wellness Center, Venice, FL 34285, USA; makisw79@yahoo.com; 5 Holy Cross Health, 4725 N Federal Hwy, Fort Lauderdale, FL 33308, USA; admin@integrafunctionalmedicine.com; 6 Food Engineering Department, Universidad de La Serena, Av. Raúl Bitrán 1305, La Serena 1700000, Chile; avegag@userena.cl; 7 Center for Cardiovascular and Nutrition Research (C2VN), Aix-Marseille University, Inserm 1263, INRAE 1260, 13005 Marseille, France; 8 Sunlight, Nutrition, and Health Research Center, 1745 Pacific Ave., Ste. 504, San Francisco, CA 94109, USA

**Keywords:** antioxidants, cancer progression, supplement, redox balance, oxidative stress

## Abstract

Supplements are widely perceived as safe and beneficial; yet in oncology, this assumption is questionable. Clinical trials over the past few decades have often produced disappointing results, raising a critical question: are these agents used correctly, or could they inadvertently cause harm? This review examines supplements frequently used in cancer care—such as oral vitamin C, berberine, N-acetylcysteine, vitamin D, vitamin E, melatonin, polyphenols, alpha-lipoic acid, selenium, and coenzyme Q10—which can act as tumor suppressors or promoters depending on dose, route, and disease stage. We examine their dual antioxidant and pro-oxidant properties, revealing that therapeutic outcomes are shaped not only by the molecule itself, but also by bioavailability, dosing thresholds, and the tumor redox environment. Building on these insights, we propose that four factors may be considered to guide clinical use: ensure that anticancer effects are not overshadowed by antioxidant activity, achieve sufficient bioavailability, confirm pro-oxidant concentrations where possible, and prioritize supplements that target the respiration-supported non-OxPhos pathways. By framing supplements as context-dependent redox modulators rather than universally beneficial agents, this review provides a mechanistically grounded framework for informing future research and safer, more effective integrative oncology strategies.

## 1. Introduction

In biology, phenomena are often presented in clear-cut divisions, yet the underlying complexities frequently reveal a spectrum of possibilities that challenge these simplified categorizations. For example, in one popular dichotomy, cells are in either an anaerobic state exclusively fermenting lactate or an aerobic state powered exclusively by oxidative phosphorylation (OxPhos). In another dichotomy, autophagy and mitophagy are always assumed to be good, yet new evidence shows that autophagy and mitophagy are sometimes used by cancer to evade apoptosis [1]. The same can be said for free radicals and antioxidants: oxidants are often considered harmful, while antioxidants are generally viewed as beneficial. Like many biological processes, redox biology is nuanced. Insufficient amounts can lead to reductive stress, while excess can result in oxidative stress and macromolecular damage. Excess reactive oxygen species (ROS) are likely partially responsible for not only carcinogenesis but also the maintenance of the malignant phenotype [2]. ROS in moderately elevated amounts can stabilize hypoxia-inducible factor (HIF) proteins regardless of oxygen content, resulting in so-called pseudo-hypoxia, and HIF stabilization can lead to the phenotypical cancer metabolic reprogramming that has now become a hallmark of cancer [3]. Due to the increases in ROS observed in cancer cells compared to normal cells, they are more dependent on endogenous antioxidant systems than normal cells [4]. Additionally, natural products containing numerous antioxidants are becoming increasingly popular among cancer patients. However, these products may have limitations when used as adjuncts to other therapies. Indeed, some antioxidants could be ineffective or even harmful to cancer patients because they might inadvertently contribute to tumor progression [5]. For example, a study shows that 90 breast cancer patients treated with megadoses of antioxidants alongside standard therapy had worse survival trends compared to those who did not receive antioxidants [6]. In this review, we examine the most commonly used cancer supplements, focusing on their paradoxical redox effects and impacts on tumor progression in vitro and in humans. We highlight compounds capable of reaching pro-oxidant levels, which may synergize with standard therapies, and explore additional mechanisms such as metabolic modulation, microbiome regulation, anti-angiogenic activity, and renin–angiotensin inhibition. The relationship between antioxidants and cancer is complex: while supplementation may offer preventive benefits, clinical results are inconsistent, and in advanced disease, exploiting pro-oxidant potential may selectively induce apoptosis in cancer cells. Factors such as dose, route of administration, absorption, and bioavailability are critical and underline that redox homeostasis, rather than antioxidant activity alone, is the true therapeutic target. We propose a framework for clinical use based on four criteria: ensuring that anticancer effects are not overshadowed by antioxidant activity, verifying sufficient bioavailability, confirming pro-oxidant concentrations when possible, and targeting the respiration-supported non-OxPhos pathways. By reframing supplements as context-dependent redox modulators, this review aims to guide future research and inform safer, more effective integrative oncology strategies.

## 2. Search Strategy and Study Selection

### 2.1. Data Sources

Electronic searches were conducted between November 2025 and April 2026 using MeSH and non-MeSH terms across two databases, PubMed and ScienceDirect. This was supplemented by searching publication reference lists (snowball procedure) and through Google Scholar, as some relevant studies were not indexed in major databases. No language or date restrictions were applied.

### 2.2. Study Selection

The included articles comprised randomized controlled trials, observational studies, narrative and systematic reviews, meta-analyses, and mechanistic in vitro and in vivo studies. The following keywords were used: antioxidants, cancer, oxidative stress, redox balance, pro-oxidant, reactive oxygen species, supplements, bioavailability, mitochondria, tumor microenvironment, and the individual names of each supplement reviewed (vitamin C, vitamin D, vitamin E, berberine, NAC, melatonin, selenium, coenzyme Q10, alpha-lipoic acid, curcumin, resveratrol, quercetin, EGCG). Studies were included if they reported on the redox, metabolic, or clinical effects of the aforementioned supplements in cancer cell lines, animal models, or human populations. No restrictions were applied regarding cancer type or disease stage. Study type (in vitro, in vivo, randomized controlled trial (RCT), systematic review, meta-analysis) is specified throughout the text and in the tables to allow readers to contextualize each finding appropriately.

### 2.3. Data Collection

One investigator extracted data on supplement type, study design, cancer model, redox outcomes, bioavailability, and clinical findings. A second author verified the extracted data. When discrepancies arose, consensus was reached through discussion among all authors.

## 3. The Dual Role of Common Supplements in Oncology

In the US, cancer patients have a high prevalence of supplement use (multivitamins/minerals), at about 70% of individuals [7], similar results to those observed in Norway [8]. The natural antioxidants most commonly used by cancer patients are berberine, oral vitamin C, N-acetylcysteine (NAC), vitamin D, vitamin E, melatonin, polyphenols (curcumin, resveratrol, quercetin, and EGCG (epigallocatechin gallate)), alpha-lipoic acid (ALA), selenium, and coenzyme Q10 [9,10]. Their use is often based on the idea that antioxidant support should be beneficial. The data summarized in Table 1 show a more complex situation. Several compounds decrease ROS production under some experimental conditions, but increase ROS production or promote apoptosis under others. This dual activity is observed for berberine, vitamin C, vitamin D, vitamin E-related compounds, polyphenols (curcumin, resveratrol, quercetin, and EGCG), ALA, melatonin, NAC, CoQ10, and selenium (see Table 1).

Antioxidants may support cellular defense, but they may also reduce ROS-dependent tumor control or, at higher ROS concentrations, induce oxidative stress and cell death. NAC, polyphenols, and vitamin E illustrate this point particularly well because they can either increase or decrease ROS depending on the cancer type and experimental setting (Table 1). A study on non-cancerous but immortalized cell lines (CHO cells) showed that ALA decreases mitochondrial ROS at 20 μM, but increases mitochondrial ROS at 100 μM [36]. The final effect depends on the molecule, dose, cancer type, disease stage, redox state, and circulating concentration of the active compound [37]. This effect could stem from various factors, such as the individual’s age, disease location, disease stage, and concentration of the active substance in the bloodstream. These findings highlight the complexity of supplement use in oncology, underscoring the importance of dosage and cancer type when considering their potential therapeutic applications. We will then review each of the most commonly used supplements individually, detailing their main anticancer actions, as well as their known antioxidant and pro-oxidant effects. Where available, we will also examine their effects in vitro, in vivo, and in humans.

### 3.1. α-Lipoic Acid (ALA)

ALA is a cofactor for five enzymes or classes of enzymes: pyruvate dehydrogenase, α-ketoglutarate dehydrogenase, the glycine cleavage system (GCS), branched-chain alpha-keto acid dehydrogenase, and the α-oxo(keto)adipate dehydrogenase. The first two are critical to the tricarboxylic acid cycle (TCA). The GCS regulates glycine concentrations [38]. Its antioxidant activity stimulates intracellular glutathione, and it can increase TCA activity, enhance mitochondrial ATP production, and reduce inflammation [39]. ALA has been shown to have anticancer effects on the following cell lines: breast, lung, colon, gastric, thyroid, skin, liver, glioma, prostate, and sarcoma [39,40]. ALA promotes cystine uptake via the Xc^−^ transporter, increasing the production of GSH, a key antioxidant for reducing oxidative stress. It activates the Nrf2 pathway, leading to the upregulation of antioxidant proteins such as HO-1, NQO1, GCLC, and Trx, thus strengthening cellular protection [41]. Additionally, ALA optimizes ATP production by mitochondria without promoting electron leakage, thus preserving the integrity of the electron transport chain and maintaining energy production [42]. ALA can induce lethal autophagy, a cellular degradation process that may be employed to eliminate tumor cells by generating mitochondrial ROS. However, a double-blind, placebo-controlled trial for patients undergoing platinum-based chemotherapy with ALA reported no significant effects in regard to the tumor reduction rate, activities of daily living, or chemotherapy-induced toxicity such as neurotoxicity and pain [43]. A randomized controlled trial in breast cancer patients studied the role of ALA in counteracting paclitaxel and doxorubicin-induced toxicities. Compared with a placebo, ALA led to a significant improvement in neuropathy grade according to the NCI-CTCAE and in the Ntx-12 score after the 9th and 12th weeks of paclitaxel treatment [44].

### 3.2. Berberine

Berberine (BBR) also illustrates the link between redox biology and mitochondrial function. BBR is a heteropentacyclic alkaloid with antidiabetic properties. It reduces cell viability, inhibits migration, and decreases inflammation in several cancer models, including breast, colon, pancreatic, gastric, liver, brain, oral, bone, skin, and prostate cancer [45,46]. Mechanistically, BBR can intercalate into mitochondrial DNA and inhibit complex I of the mitochondrial respiratory chain. This promotes mitochondrial dysfunction, electron leakage, and mitochondrial superoxide production [47]. The electron leakage and increased ROS activate signaling pathways such as ASK1/JNK, stabilize the p53 protein, and induce mitochondrial membrane permeabilization, releasing cytochrome c (Cyt-c) and triggering intrinsic apoptosis via the caspase cascade [48].

### 3.3. Coenzyme Q10

Coenzyme Q10 (CoQ10) is a component of the mitochondrial electron transport chain, where it plays a role in oxidative phosphorylation, a process required for the biosynthesis of adenosine triphosphate (ATP), the primary energy source of cells. It is a potent antioxidant that can stimulate OxPhos, induce apoptosis, exhibit anti-angiogenic effects, and inhibit DNA damage [49]. CoQ10 participates in the electron transport chain as an electron carrier between complexes I, II, and III, maintaining efficient energy production and preventing electron leakage, which could generate ROS [50]. It recycles electrons in complexes I and III, contributing to a continuous antioxidant cycle and the optimal management of free radicals. CoQ10 also helps regulate the mitochondrial permeability transition pore (mPTP), reducing the release of ROS and Cyt-c, both of which are involved in apoptosis. It activates the Nrf2 pathway, a signaling pathway that promotes the expression of antioxidant genes, thereby enhancing cellular defense against oxidative stress [51]. Under certain conditions, especially with excess CoQ10 or mitochondrial dysfunction, the semi-quinone cycle of CoQ10 (CoQ10•^−^ → O_2_•^−^) can lead to the production of superoxide radicals (O_2_•^−^), amplifying oxidative stress [14]. However, one study showed that, in postmenopausal women diagnosed with cancer, higher concentrations of CoQ10 were associated with an increased risk of cancer [52].

### 3.4. Melatonin

Melatonin, also known as 5-Methoxy-N-acetyltryptamine, is a hormone that has shown anticancer effects on the following cell lines: breast, prostate, ovarian, cervical, endometrial, renal, lung, gastric, pancreatic, colon, liver, skin, sarcoma, leukemia, and oral cancer [53]. Melatonin activates the cellular defense pathway, leading to an increase in the levels of GSH, SOD, Nrf2, and HO-1, which are essential for reducing oxidative stress [54]. It activates the SIRT1/PGC-1α pathway, promoting mitochondrial biogenesis and decreasing mitochondrial ROS production, thereby reducing oxidative stress at the mitochondrial level [55]. It inhibits the action of Bcl-2, which facilitates mitochondrial permeability, the release of ROS and Cyt-c, and the activation of the intrinsic apoptotic pathway [56]. Melatonin also reduces the activity of HIF-1α [57,58]. Melatonin shows paradoxical results, with either improved survival [59,60,61,62] or no significant effects [63,64,65]. A systematic review and meta-analysis suggest the therapeutic potential of melatonin alone or in combination with adjuvant treatment [66,67,68]. However, a low level of melatonin was found to increase breast cancer risk in a one systematic review and meta-analysis [69].

### 3.5. NAC

NAC is a good example of this ambiguity. It is a potent antioxidant, can modulate immune responses, and may inhibit ferroptosis [70,71]. Its main antioxidant action is linked to glutathione (GSH) synthesis. NAC is converted into L-cysteine, which combines with L-glutamate to form GSH. This mechanism can protect cells from oxidative damage. At higher doses, however, NAC may become a pro-oxidant. By reducing ferric iron (Fe^3+^) to ferrous iron (Fe^2+^), it can promote Fenton chemistry and hydroxyl radical formation in the presence of H_2_O_2_ [72]. The same compound may therefore reinforce antioxidant defenses or contribute to oxidative cell death, including apoptosis or ferroptosis, depending on redox conditions and iron availability.

### 3.6. Polyphenols

Polyphenols from plants display a broad spectrum of anticancer properties, including antioxidant effects, metabolism regulation, and anti-angiogenic and anti-metastatic activities [73]. They showed an inhibitory effect on prostate cancer cell proliferation (AT-2 and MAT-LyLu cells), motility, and cellular competence for gap junctional communication [74]. The authors point out that these phenolic compounds found in leaves may exert the observed chemopreventive and anti-carcinogenic effects through synergic effects on oxidative stress and ROS-dependent intracellular signaling.

#### 3.6.1. Curcumin

Curcumin, a beta-diketone with two aromatic rings united by 7 carbons, is an antioxidant and anti-inflammatory, induces autophagy, inhibits angiogenesis and metastasis, causes epigenetic modifications, and alters mitochondrial energy metabolism [75]. Curcumin has shown anticancer effects on the following cell lines: breast, lung, pancreatic, brain, leukemia, and prostate cancer [76]. Curcumin directly neutralizes various free radicals (such as •OH, O_2_•^−^, ROO•, ONOO^−^, and ^1^O_2_) through its phenolic groups and β-diketone structure [77]. It activates the Keap1/Nrf2/ARE pathway by alkylating the cysteine residues of Keap1, causing its dissociation and allowing the translocation of Nrf2 into the nucleus [78]. Curcumin chelates metals like Fe^2+^, Cu^2+^, and Zn^2+^, preventing the Fenton reaction and the generation of hydroxyl radicals [79]. Curcumin generates H_2_O_2_ in culture media, a pro-oxidant mechanism observed under in vitro conditions. It inhibits anti-apoptotic proteins such as Bcl-2 and Bcl-xL, facilitating the release of Cyt-c and activating the intrinsic apoptosis pathway through caspase-9 and caspase-3 [77]. In a tumor context, curcumin inhibits Nrf2, impairing the antioxidant resistance of cancer cells and making them more vulnerable to oxidative stress [80]. Curcumin also inhibits HIF-1α, a key factor in hypoxic adaptation, inducing oxidative stress and limiting tumor growth [81]. Curcumin may also be involved in overcoming paclitaxel resistance. Curcumin has great potential in targeting P-gp, an ATP-binding cassette transporter that can remove chemo-therapeutic agents from cancer cells. This inhibition of P-gp can lead to the accumulation of paclitaxel inside cancer cells, which, in association with curcumin, can significantly enhance ROS generation [82]. Curcumin increases survival [83,84,85].

#### 3.6.2. EGCG (Epigallocatechin Gallate)

EGCG is a polyphenol and catechin found in high concentrations in green tea, cocoa, and various fruits. EGCG has three aromatic rings connected by a pyran ring. Its antioxidant properties are conferred by the transfer of hydrogen atoms or single-electron transfer involving its hydroxyl groups. EGCG inhibits carcinogen activity, tumorigenesis, proliferation, and angiogenesis and induces apoptosis. It has shown anticancer effects in the following cell lines: breast, colorectal, endometrial, gastric, glioblastoma, liver, lung, melanoma, multiple myeloma, nasopharyngeal, neck, ovarian, pancreatic, and prostate [86]. EGCG exhibits antioxidant properties and can modulate cellular redox balance; it can scavenge ROS and enhance the activity of antioxidant enzymes, such as superoxide dismutase and catalase.

#### 3.6.3. Quercetin

Quercetin is a polyphenol that exerts anticancer effects by modulating cell cycle progression, inhibiting cell proliferation, promoting apoptosis, and inhibiting angiogenesis and metastasis progression, while also affecting autophagy [87]. Quercetin has shown anticancer effects on the following cell lines: lung, skin, brain, colon, breast, prostate, ovarian, gastric, liver, bone, and oral cancer [88]. It chelates metal ions (Fe^2+^/Cu^2+^) via its 3-OH and 4-oxo groups, blocking the Fenton reaction, which is responsible for generating hydroxyl radicals [89]. Quercetin activates the Nrf2 signaling pathway, increasing the expression of antioxidant genes such as HO-1, NQO1, SOD, and CAT, thereby strengthening the cellular antioxidant defenses [90]. Quercetin inhibits the isoforms NOX2 and NOX4, reducing the production of superoxide ions (O_2_•^−^) and thus mitigating oxidative stress [91].

#### 3.6.4. Resveratrol

Resveratrol is a polyphenol found in blueberries and grapes, among other fruits. It exerts different anticancer effects such as anti-inflammatory activity, antioxidant activity, apoptosis promotion, EMT regulation, metastasis inhibition, angiogenesis inhibition, and regulation of cell metabolism, including mitochondrial activity [92,93]. Resveratrol has shown anticancer effects on these cell lines: breast, esophageal, colon, lung, pancreatic, gastric, liver, neuroblastoma, brain, sarcoma, leukemia, skin, and prostate cancer [94,95]. By activating the SIRT1 → PGC-1α pathway, resveratrol induces mitochondrial biogenesis, reducing mitochondrial ROS levels and increasing the activity of antioxidant enzymes such as SOD and CAT [96]. In terms of metal chelation, resveratrol inhibits copper, thereby reducing the effects of the Fenton reaction and limiting hydroxyl radical generation [97]. Moreover, the increase in mitochondrial ROS promotes cell death. Resveratrol also inhibits mitochondrial respiratory chain complexes, causing electron leakage and increased production of O_2_•^−^ in the mitochondria [98], inducing oxidative stress specifically in tumor cells.

### 3.7. Selenium

The anticancer effects of low-dose selenium include antioxidant activity, promoting DNA repair, inducing apoptosis, inhibiting angiogenesis, inducing cell cycle arrest, and stimulating the immune system [99]. Selenium has shown anticancer effects on the following cell lines: breast, lung, thyroid, liver, and prostate [99]. The different isoforms of GPx use selenium to reduce peroxides such as H_2_O_2_ (hydrogen peroxide) and organic hydroperoxides (ROOH), thus contributing to the reduction in oxidative stress [100]. GPx4 suppresses ferroptosis by reducing lipid peroxides, and Se is essential for its activity [101]. At high doses, selenite can be reduced by TrxR, generating redox cycles and reactive oxygen species (O_2_•−/H_2_O_2_), leading to oxidative stress that can provoke apoptosis [102]. Selenium does not show clinical benefits (see Table 2).

### 3.8. Vitamin C

Vitamin C or ascorbic acid is a water-soluble compound found in fruits and vegetables that is involved in tissue repair, collagen formation, and the enzymatic production of some neurotransmitters [103]. It exerts antioxidant effects and pro-oxidative cytotoxicity, and induces anticancer epigenetic regulation, immune modulation, and inhibition of cancer cell metabolism [104]. Vitamin C has shown anticancer effects on the following cell lines: breast, colon, pancreatic, skin, lymphoma, leukemia, renal, liver and lung [105]. These reactions enable vitamin C to reduce oxidative stress by neutralizing ROS [106]. Vitamin C also protects DNA by reducing the levels of 8-OHdG, a marker of DNA oxidation, and it regenerates metal ions such as Fe^3+^ and Cu^2+^, converting them into their active reduced forms. As a pro-oxidant, vitamin C can induce selective cytotoxicity in tumor cells. By reducing Fe^3+^ to Fe^2+^, ascorbate drives the Fenton reaction [107], generating hydroxyl radicals (•OH) from extracellular H_2_O_2_. This H_2_O_2_ can penetrate tumor cells, particularly those deficient in catalase, thereby increasing oxidative stress and cellular damage. Vitamin C may also interfere with the regulation of HIF-1α [108], a key factor in angiogenesis and the adaptation of tumor cells to hypoxic conditions. Some studies have reported improved survival [109,110,111,112,113,114], while others have found no effect [115,116]. However, the studies reporting improved survival involved the use of intravenous vitamin C, thus indicating pro-oxidant effects, while studies using oral vitamin C, and thus exhibiting antioxidant effects, reported no effect on survival.

### 3.9. Vitamin D

Vitamin D is a prohormone produced in the skin from UVB irradiance on 7-dehydrocholesterol followed by a thermal reaction or ingested from supplements or food. It is hydrolyzed in the liver to become 25-hydroxyvitamin D [25(OH)D], the main circulating metabolite. This metabolite can be further hydrolyzed in the kidneys or other organs as needed to form 1,25-dihydroxyvitamin D (calcitriol), which can help to fight cancer in organs [117]. Calcitriol binds to vitamin D receptors on cell nuclei, where they can upregulate and downregulate certain genes [118]. Vitamin D, a fat-soluble compound, can inhibit tumor cell proliferation, angiogenesis, hormones, EMT, and cancer cell metabolism, and induce apoptosis, autophagy, and cell differentiation; target cancer stem cells; enhance the immune system; regulate inflammation; influence the microbiome; and modulate the renin–angiotensin system [118,119]. Vitamin D, via its receptor (VDR), activates the Nrf2 signaling pathway, which induces the transcription of several genes involved in antioxidant defense mechanisms. This includes the activation of HO-1 (heme oxygenase), and enzymes necessary for glutathione biosynthesis (GCLC/GCLM) [120]. This activation promotes the production of GSH, a powerful antioxidant that helps neutralize ROS. In addition, vitamin D increases the activity of antioxidant enzymes such as GPx, glutathione reductase (GR), SOD2, and CAT, all of which contribute to cellular protection against oxidative stress [121]. Furthermore, vitamin D inhibits NOX2 and NOX4, which are responsible for generating ROS, thereby limiting their production and preserving cellular integrity [122]. VDR overexpression is associated with the promotion of oxidative stress. After VDR knockdown, antioxidant levels (SOD2, PRDX5, GPX1, and GCLM) increase significantly, suggesting that active VDR suppresses these antioxidants in this tumor context, fostering a pro-oxidant environment that stimulates cancer cell proliferation [123]. While vitamin D has been shown to have antioxidant effects in mouse models [124] and on breast, colon, and head and neck cancer cell lines [32], the many other mechanisms by which vitamin D can affect the risk of cancer greatly outweigh the antioxidant effects. Indeed, the antioxidant effect of high-dose vitamin D3 supplementation appeared weak and statistically insignificant in a weaning pig study, with only marginal improvements observed in specific markers such as superoxide dismutase (SOD) activity and malondialdehyde (MDA) levels, but no significant change in total antioxidant capacity (T-AOC) [125]. Vitamin D appears to impact survival [126], its effects may operate through mechanisms beyond its antioxidant activity. The evidence comes from several types of studies: geographical ecological studies, prospective cohort studies related to serum 25-hydroxyvitamin D [25(OH)D] concentrations, RCTs, and mechanistic studies [118]. The effect of vitamin D on cancer risk and progression is related to serum 25(OH)D concentration, not vitamin D dose. Observational studies can show dose–response relationships over large 25(OH)D ranges (e.g., for breast cancer [127]). Vitamin D RCTs tend to be poorly designed, conducted, and analyzed: typically, participants have high 25(OH)D concentrations, those in the treatment arm are given low vitamin D doses, those in the control arm are also permitted to take vitamin D, and the results are analyzed according to intention to treat rather than achieved 25(OH)D concentration [128]. Nonetheless, the largest vitamin D RCT to date that assessed the effect of 2000 IU/day vitamin D supplementation on the risk of cancer found that participants with mean 25(OH)D concentrations ~78 nmol/L and BMIs < 25 kg/m^2^ had a 25% reduction in all-cancer incidence [129]. Participants with higher BMIs did not have reduced cancer incidence rates. In addition, there was a 25% reduction in all-cancer mortality rate after censoring the first one or two years of data. In general, the effect of vitamin D on cancer risk is higher for mortality rate than incidence rate [118]. A meta-analysis of 10 randomized controlled trials concluded that, while vitamin D supplementation did not significantly reduce overall cancer incidence, the primary benefit of vitamin D supplementation appears to lie in reducing cancer mortality [126]. Another recent study based on data from three randomized controlled trials showed that vitamin D can prevent 30 000 cancer deaths per year in Germany (13% of the total) [130].

### 3.10. Vitamin E

Vitamin E is another case in which the chemical form matters. Vitamin E includes α-, β-, γ-, and δ-tocopherols (α-T, β-T, γ-T, δ-T) and α-, β-, γ-, and δ-tocotrienols (α-T3, β-T3, γ-T3, δ-T3). It has antioxidant and anti-inflammatory properties, but can also promote apoptosis [131]. Tocotrienols, especially γ-T3 and δ-T3, have stronger antioxidant activity than α-T due to their unsaturated chain, which improves their distribution in lipid membranes. α-tocopherol inhibits NADPH oxidase (NOX) [132], thereby reducing the extracellular production of O_2_•^−^. This may explain some of the clinical side effects associated with high vitamin E consumption. δ-tocotrienol inhibits HMG-CoA reductase, reducing mevalonate synthesis, which in turn decreases the isoprenylation of K-Ras protein, inhibiting the proliferation of ROS-dependent tumor cells [133]. Tocotrienols, especially γ-T3, activate death receptors (DR4 and DR5), promoting extrinsic apoptosis in cancer cells. Vitamin E inhibits the JAK2/STAT3 pathway, reducing anti-apoptotic defenses and sensitizing tumor cells to pro-apoptotic ROS [134]. Additionally, it inhibits the Akt/mTOR pathway, reducing cell survival and facilitating apoptosis in synergy with ROS. Vitamin E does not seem to show clinical benefits and might even be detrimental regarding mortality [135]. In brief, the results for vitamin E have been clearly disappointing in large-scale human trials to date (see Table 1 [136]). However, the outcomes depend on the nutritional status of the population and the form and dose of vitamin E used. Supplementation with γ-T, δ-T, and tocotrienols, but not α-T, could reduce cancer risk [136].

It is generally accepted that antioxidants have beneficial effects, particularly for cancer prevention, although their potential effects as adjuncts to treatments and on mortality should also be considered. However, RCTs, systematic reviews, and meta-analyses show that, in general, antioxidants have disappointing results, except for vitamin D, melatonin, and vitamin C taken orally, and with vitamin C primarily obtained from food rather than supplements (see Table 2).

**Table 2:** Summary of effects of supplements in cancer patients: a review of randomized controlled trials and systematic reviews and meta-analyses.

Source	Clinical Context	Patients (*n*)	Study Type	Subjects	Antioxidants	Dosage	Main Findings
[137]	Prevention	29,133	RCT	Male smokers cancer-free	Alpha-tocopherol and beta-carotene supplementation	Daily supplementation with b-carotene (20 mg) and/or vitamin E (50 mg dl a-tocopheryl acetate)	No protective effect of vitamin E or beta-carotene supplementation against cancers of the upper aerodigestive tract
[135]	Prevention	232,606	Systematic review and meta-analysis	Men and women for primary and secondary prevention	Beta-carotene, vitamins A, C, E, and selenium (alone or in combination)	Daily or on alternate days supplementation with beta carotene 1.2 to 50.0 mg (mean, 17.8 mg); vitamin A 1333 to 200 000 IU (mean, 20 219 IU); vita- min C 60 to 2000 mg (mean, 488 mg), vitamin E 10 to 5000 IU (mean, 569 IU); and selenium 20 to 200 μg (mean 99 μg).	Treatment with beta carotene, vitamin A, and vitamin E may increase mortality; the majority of the studies are for secondary prevention (patients who already have cancer).
[138]	Prevention	170,525	Systematic review and meta-analysis	Prevention of gastrointestinal cancers (not specified)	Beta-carotene, vitamins A, C, E, and selenium (alone or in combination)	Daily or on alternate days supplementation with beta-carotene (15–50 mg), vitamin A (1.5–15 mg), vitamin C (120–2000 mg), and vitamin E (30–600 mg), and selenium (50–228 μg).	No evidence that antioxidant supplements can prevent gastrointestinal cancers. Antioxidants increase overall mortality.
[139]	Prevention and mortality	104,196	Systematic review and meta-analysis	Men and women for primary cancer	Beta carotene, selenium, vitamin C (ascorbic acid), vitamin E (α-tocopherol), and lycopene alone or in combination	Beta-carotene 15–30 mg/day; vitamin C 120–250 mg/day; vitamin E 30–900 IU/day; and selenium 100–200 µg/day.	Antioxidants did not significantly reduce total cancer mortality.
[140]	Prevention	161,045	Systematic review and meta-analysis	Men and women cancer free	Beta-carotene, vitamin A, vitamin C, vitamin E (α-tocopherol), and selenium	Vitamin A (15 mg or 10 000 to 300 000 IU, daily or weekly); vitamin C (120, 180, or 250 mg, daily); vitamin E (30 to 600 mg or 60 to 600 IU, daily or on alternate days); beta-carotene (6 to 75 mg; daily or on alternate days); and selenium (50 to 200 µg, daily).	No clinical evidence to support an overall primary and secondary preventive effect of antioxidant supplements on cancer.
[141]	Prevention	18,314	RCT	Men and women at risk of lung cancer	Beta-carotene and retinyl palmitate	Daily supplementation with 30 mg beta-carotene and 25,000 IU retinyl palmitate	28% more cancer incidence in the experimental arm.
[142]	Prevention	NS	Systematic review and meta-analysis	NS	Beta-carotene	Beta-carotene 6 to 50 mg	Beta-carotene increased the risk of lung cancer.
[66,67]	Treatment outcomes & Mortality	NS	Umbrella review of meta-analysis and systematic review and meta-analysis	Cancer patients	Melatonin	Daily supplementation, 0.05 to 40 mg/day.	Increased survival at one year.
[143]	Prevention	27,232	Systematic review and meta-analysis	Men and women cancer free	Selenium supplementation	Daily supplementation, 200 µg to 500 μg/day of selenium.	Selenium supplementation had no significant impact on the risk of developing cancer or on cancer-related mortality.
[144]	Prevention and mortality	29,584	RCT	Healthy men and women at increased risk of developing esophageal cancer and gastric cancer	Selenium, vitamin E, and beta-carotene	Daily supplementation of 50 μg selenium, 30 mg vitamin E (alpha-tocopherol), and 15 mg beta-carotene.	No effect on prevention, reduction of mortality.
[145]	Supportive care	NS	Systematic review	Cancer patients	Vitamins, minerals, phytochemical compounds and amino acids compounds	Daily supplementation 20 to 40 mg/day of melatonin. NS for the other supplements.	No evidence suggesting that taking antioxidant supplements alongside cancer therapy was harmful.
[146]	Treatment outcomes	17,062	Systematic review and meta-analysis	Breast cancer patients	Vitamin A, C, or E	NS	Only vitamin C increased survival.
[147]	Prevention	NS	Systematic review and meta-analysis	Healthy men and women	Vitamin A, vitamin C, vitamin D, vitamin E (alpha-tocopherol), lycopene, folate, iron, carotenoids, beta-carotene, selenium, pyridoxine	Vitamin A, 1500 to 8000 µg/day; vitamin C, 310 to 750 mg/day; vitamin D, 125 to 620 IU/day; vitamin E, 40 to 300 IU/day; folate 128 to 703 µg/day; beta-carotene 20 mg/day, lycopene, 837 to 11 680 µg/day; iron, <11.3 to >17.3 mg/day or 48 to 336 µg/1000 kcal; α-carotene, 192 to 1561 µg/day; lutein/zeaxanthin 885 to >2072 µg/day; β-cryptoxanthin, 75.7 to >193.5 µg/day; selenium, NS; pyridoxine, NS.	None of the studied associations show a significant effect on the risk of non-Hodgkin lymphoma for vitamin A, vitamin C, vitamin D, vitamin E, or lycopene intake.
[148]	Prevention	12,741	RCT	Men and women cancer-free	Vitamin C, vitamin E (α-tocopherol), beta carotene, selenium, and zinc, or a placebo.	Daily capsule of a combination of 120 mg of ascorbic acid, 30 mg of vitamin E (unspecified), 6 mg of beta carotene, 100 µg of selenium, and 20 mg of zinc, or a placebo.	No beneficial effects of antioxidant supplementation in men and women in the long term (5-year postintervention period).
[149]	Prevention	14,641	RCT	Men	Vitamin E (synthetic α-tocopherol) every other day and 500 mg vitamin C daily.	Individual supplements of 400 IU of vitamin E every other day and 500 mg vitamin C daily.	Vitamin E and C supplementation do not reduce the risk of prostate or total cancer.
[150]	Prevention	NS	Systematic review and meta-analysis	Men and women cancer-free	Vitamin C	NS	Vitamin C-rich foods are associated with a reduced risk of breast cancer, colorectal cancer, and prostate cancer, but no such benefit was found with vitamin C supplements.
[151]	Prevention	62,619	Systematic review and meta-analysis	Men and women cancer-free	Vitamin C	120 to 500 mg/day	No evidence to support the use of vitamin C supplements for prevention of cancer.
[152]	Prevention	NS	Umbrella review	Men and women cancer-free	Vitamin C	50 to >1000 mg/day	Vitamin C-rich foods are associated with a lower risk of bladder cancer, breast cancer, cervical neoplasms, endometrial carcinoma, esophageal cancer, gastric cancer, glioma, lung cancer, pancreatic cancer, prostate cancer, and renal cell cancer.
[153]	Prevention and mortality	26,347	Systematic review and meta-analysis of observational studies	Women with and without breast cancer	Vitamin C	55 to >1000 mg/day	High dietary vitamin C intake is associated with reduced breast cancer incidence and mortality; no significant preventive effect has been observed with supplementation.
[154]	Prevention	NS	Systematic review and meta-analysis	Men and women cancer-free	Vitamin C, carotenoids, and vitamin E	Vitamin C: 39 to 388 mg/day; Vitamin E: 4.9 to 21 mg/day; β-carotene: 679 to 15,034 µg/day; α-carotene: 43.6 to 696 µg/day; β-cryptoxanthin: 91.4 to 1418 µg/day; Lutein: 221 to 1723 µg/day; Zeaxanthin: 40.5 to 261 µg/day; Lycopene: 622 to 4047 µg/day.	Higher dietary intake and/or blood concentrations of vitamin C, carotenoids, and α-tocopherol were associated with reduced risk of total cancer.
[155]	Treatment outcomes	NS	Systematic review and meta-analysis	Colorectal cancer patients	Vitamin D	400 IU/day to 4000 IU/day or 100,000 IU/4 months	30% reduction in adverse CRC outcomes with vitamin D supplementation.
[126]	Prevention and mortality	75,239	Systematic review and meta-analysis of RCTs	Men and women general population	Vitamin D	400 IU/day to 3279 IU/day	No effect on cancer incidence; significant reduction in cancer mortality.
[156]	Prevention	39,876	RCT	Healthy US women	Vitamin E (α-tocopherol)	600 IU of natural-source vitamin E (α-tocopherol)	No benefit for cancer prevention.

NS: Not Specified; RCT: Randomized Controlled Trial.

The evidence summarized in this table is notably inconsistent, reflecting the complexity of antioxidant biology in cancer. Vitamin C supplementation shows no effect on cancer prevention in RCTs, despite strong epidemiological signals from dietary studies. This discrepancy likely arises because dietary sources of vitamin C include many other nutrients that may contribute to the observed effects [157]. Similarly, vitamin E supplementation produces conflicting results depending on population, dose, and cancer type, with no clear protective effect emerging from large trials. The main problem with most vitamin E RCTs was that only α-tocopherol was used. The most consistent and concerning finding is that beta-carotene supplementation significantly increases lung cancer risk in high-risk populations, and that combinations of antioxidant supplements may increase overall mortality. Vitamin D appears to stand out, with evidence suggesting reduced cancer mortality and improved colorectal cancer outcomes, although results vary according to dosing strategy. Vitamin D has been found in RCTs to significantly reduce all-cancer incidence for participants with BMI < 25 kg/m^2^ and all-cancer mortality rates [129]. A meta-analysis of vitamin D RCTs found a significant reduction in all-cancer mortality rates but not incidence rates [126]. The main problem with vitamin D RCTs is that participants generally have high serum 25(OH)D concentrations. Those in the treatment group are given low vitamin D doses; those in the control group are also given low vitamin D doses and permitted to take additional vitamin D, and the results are analyzed according to treatment rather than achieved 25(OH)D concentration. As a result, observational studies of vitamin D and health outcomes provide the best evidence [118,158]. Overall, the discrepancy between the apparent benefits of food-derived antioxidants and the neutral or harmful effects of isolated supplementation remains unexplained. Dose is an important determinant of efficacy, and some of the studies listed in Table 2 use widely different doses. However, dose alone is not sufficient in a system where transporters can become saturated and/or obesity may act as a limiting factor for some supplements. To summarize, these in vitro and clinical observations raise an immediate practical question: can these compounds achieve the concentrations necessary to exert pro-oxidant effects in vivo?

## 4. Absorption and Bioavailability

The answer depends critically on absorption and bioavailability—the pharmacological bridge between a supplement’s theoretical potential and its actual effect in tumor tissue. Below, we summarize the absorption rates for each supplement, as these data are central to interpreting both experimental and clinical findings.

### 4.1. Group 1—Sufficient Absorption, and Theoretically Achievable Pro-Oxidant Concentrations via Oral Administration

The following supplements share sufficiently high oral absorption rates to theoretically reach biologically active—and potentially pro-oxidant—concentrations in systemic circulation. Vitamin D absorption ranges from 62% to 100% [159,160]. Logically, the dose plays an important role, and the same applies to vitamin D, where high doses (8000 IU/day) produce more significant effects compared to standard doses (400 IU/day) [161]. However, high doses are not always sufficient in obese individuals, as excess adipose tissue can sequester vitamin D [119]. In a study with 12 healthy volunteers, single doses of racemic ALA were administered orally at a dose of 200 mg, with absorption rates ranging from 20% to 38% [162]. The absorption of selenium is very efficient, ranging from >50% for selenite to 100% for selenate, and can exceed 90% for organic forms [163]. The absorption of β-carotene seems to vary significantly, ranging from 5% to 65%, influenced by factors such as diet, genetic traits, and the individual’s health condition [164]. This wide variability makes individual predictions difficult and limits its reliable classification as a consistently pro-oxidant supplement.

### 4.2. Group 2—Intermediate or Paradoxical Absorption via Oral Administration

The oral absorption of vitamin C is highly regulated. The higher the oral intake of vitamin C, the more the vitamin C transporters (saturable, SVCT1/2) become saturated. Indeed, after a single dose of 200 mg of vitamin C taken orally, the absorption rate is 100%, whereas it drops to 33% for a single dose of 1250 mg [165]. A scoping review study showed that liposomal oral vitamin C improved Cmax by 1.2 to 5.4 times and AUC by 1.3 to 7.2 times [166], which is still far from the levels achieved with intravenous administration. Indeed, intravenous vitamin C can achieve plasma concentrations of 23 to 49 mM, which is 60 to 270 times higher than those obtained with oral administration (whether liposomal or conventional) [166,167,168]. For doses of melatonin ranging from 2 to 4 mg, the absorption is approximately 15% [169]. A systematic review analyzed oral doses ranging from 0.3 mg to 100 mg, and the absorption varied between 9% and 33% [170]. Resveratrol comes in several forms. Trans-resveratrol appears to be the more stable natural form. Resveratrol presents a unique paradox: its oral absorption is approximately 75% and occurs primarily through transepithelial diffusion, yet extensive metabolism in the intestine and liver results in a systemic bioavailability of the free form that is significantly less than 1% [171]. Despite its low bioavailability, resveratrol shows efficacy in vivo. This may be explained by the conversion of both sulfates and glucuronides back to resveratrol in target organs such as the liver. Another possible explanation could be the enterohepatic recirculation of resveratrol metabolites, followed by its deconjugation in the small intestine and its re-absorption. Finally, in vivo effects could be explained by the activity of its metabolites [172]. Vitamin E presents an intermediate profile, with oral absorption ranging from 10% to 33% [173].

### 4.3. Group 3—Oral Bioavailability Too Low to Achieve Pro-Oxidant Effects

For the following compounds, oral bioavailability was too limited to expect pro-oxidant concentrations in systemic circulation. The absorption of berberine is very low (<1%) [174]. However, some publications have demonstrated that berberine’s bioavailability can be improved using technologies such as MLDH (MgAl monolayer hydrotalcite). The goal is to promote the solubility and bioavailability of berberine [175]. CoQ10 has relatively low oral bioavailability in humans—less than 2% for standard forms [176]—due to its high lipophilicity (fat-solubility), large molecular weight, and poor water solubility. Absorption is slow and incomplete, with significant inter-individual variability [177]. Curcumin is a problematic case. Even with the best formulations, the plasma levels of free (non-conjugated) curcumin remain 1000 times lower than the concentrations used in vitro to demonstrate biological effects [178]. EGCG has low oral bioavailability in humans, with levels in plasma reaching less than 1% of the ingested dose. Much of the compound undergoes first-pass metabolism (glucuronidation, sulfation, methylation) in the intestine and liver [179]. For an oral dose of 400 mg of NAC, the absorption is 4% [180]. Another study reported absorption rates ranging from 6% to 10% [181]. Quercetin’s bioavailability is higher than that of EGCG, but still relatively low (<10%) due to its poor water solubility. Most absorbed quercetin undergoes extensive first-pass metabolism in the intestine (by β-glucuronidase) and liver, appearing in the plasma primarily as conjugated metabolites (glucuronides, sulfates, and methylated forms) [182]. For example, quercetin aglycone has been shown to interact with certain receptors, particularly the aryl hydrocarbon receptor, which is involved in the development of cancers [183]. The observed anticancer effects of these supplements in vitro therefore likely reflect mechanisms other than direct redox modulation, or require alternative delivery strategies.

These absorption constraints have direct clinical implications. Supplements in Group 1—selenium, vitamin A, vitamin D, and ALA—are the most plausible candidates for theoretically achievable pro-oxidant concentrations. For compounds in Groups 2 and 3, achieving pro-oxidant concentrations via oral administration is unlikely without alternative formulations or intravenous delivery, as exemplified by vitamin C. This distinction should inform both clinical decision making and trial design. However, bioavailability is only one piece of the puzzle. Even when a supplement reaches systemic circulation, its ultimate effect depends on where and how it interacts with cancer cell metabolism—particularly at the mitochondrial level.

## 5. Mitochondrial Respiration and Supplements

In many cancers, abnormalities in mitochondrial structure, number, or function have been highlighted [3,184]. From a mitochondrial perspective, cancer results from chronic alterations in OxPhos [185]. Like all cells, cancer cells require energy to survive and primarily produce their ATP through two pathways: cytosolic substrate-level phosphorylation (cSLP) and mitochondrial substrate-level phosphorylation (mSLP) [184,185,186]. Cancer cells rely on only a small fraction (~2–7%) of their mitochondrial oxygen capacity to maintain the respiratory flux required for biosynthetic redox processes [187]. This is consistent with oxygen consumption within mitochondria. Mitochondrial respiration is responsible for more than 90% of oxygen consumption in humans. Cells utilize oxygen as the final electron acceptor in the aerobic metabolism of glucose to generate ATP, which fuels most active cellular processes [188]. However, a recent study highlights the complexity of the mitochondrial ‘problem’. Although OxPhos (and thus ATP production) may be impaired, non-OxPhos mitochondrial pathways that rely on oxygen as the terminal electron acceptor in the ETC can support tumor progression [189]. This article highlights numerous respiration-supported non-OXPHOS pathways such as (i) de novo pyrimidine synthesis via dihydroorotate dehydrogenase (DHODH); (ii) aspartate provision by mitochondrial Glutamate–Oxaloacetate Transaminase 2 (GOT2); (iii) mitochondrial one-carbon metabolism via serine hydroxymethyltransferase 2 and methylenetetrahydrofolate dehydrogenase 2; (iv) nicotinamide nucleotide transhydrogenase (NNT) using protonmotive force to supply mitochondrial NADPH; (v) glycerol-3-phosphate shuttling; (vi) the TCA cycle; (vii) heme and Fe-S cluster synthesis; (viii) proline and hydroxyproline metabolism; (ix) H_2_S oxidation; and (x) choline oxidation [189]. We summarize the effects of supplements on respiration-supported non-OxPhos mitochondrial pathways in Table 3.

Based on this perspective on targeting respiration-supported non-OxPhos pathways, berberine, curcumin, EGCG, vitamin D, and ALA would appear to inhibit some pathways, thereby potentially reducing their support for cellular proliferation. In contrast, CoQ10, resveratrol, melatonin, selenium, NAC, and vitamin E might activate them, possibly promoting proliferation. Finally, vitamin C would exhibit a well-known ambivalent, dose-dependent effect. These assignments are based on available in vitro and mechanistic data and should be interpreted as hypothesis-generating rather than as clinical guidance. The effectiveness of supplements could therefore be context-dependent, dose-dependent, and determined by which respiration-supported non-OxPhos pathway is dominant in the specific tumor. This would allow us to understand why many supplements have been mentioned as potentially involved in tumor progression, in both in vitro and in vivo models, which we will discuss further in the next section. This metabolic heterogeneity would have direct consequences for how supplements might behave in a tumor context. Indeed, activating certain mitochondrial pathways may not always be desirable: under some conditions, the same supplements that support normal cell function could inadvertently fuel tumor progression.

## 6. Biological and Biochemical Mechanisms of the Effects of Antioxidant Supplements on Tumor Progression

This apparent paradox—of supplements promoting cancer survival rather than cell death—becomes less surprising when one considers the specific biochemical environment of advanced tumors. The antioxidant activity of GSH drives metastasis in breast cancer [212]. Indeed, GSH is responsible for tumor progression and chemoresistance [213]. A study on 460 women with breast cancer showed that excess GSH was responsible for chemotherapy resistance [214]. This activity may partly result from glutaminolysis, which supports cancer cell maintenance and survival by providing NADPH and GSH, thereby compensating for the reduced energy production from OxPhos and mitigating ROS under normal cancer cell conditions [208]. A study showed that NAC and vitamin E enhance tumor cell proliferation in both mouse and human lung tumor cells by reducing ROS, DNA damage, and p53 expression [215]. Additionally, NAC and vitamin C can stimulate angiogenesis during KRAS-driven lung cancer progression. These antioxidants increase the expression of BACH1 and HIF1α, which promote the transcription of VEGFs, VEGF receptors, and NRPs [216]. Similar results are observed in melanoma, where NAC and the soluble vitamin E analog Trolox increase metastasis in mice [217]. Furthermore, antioxidants stabilize BACH1, leading to the activation of glycolysis-related genes like HK-II and GAPDH. This overexpression results in increased glucose uptake, higher glycolysis rates, and greater lactate secretion, contributing to enhanced metastasis in lung cancer in mice [218]. Low-dose vitamin C and niacin (vitamin B3) lines increase the proliferation of CSCs in colon cancer cell lines, and the effect is reversed with a high concentration of vitamin C [219]. An in vivo study on melanoma demonstrated that low concentrations of vitamin C promoted tumor growth. The findings suggested that a lower dose of vitamin C (equivalent to 0.5 g/70 kg) administered orally led to an increase in melanoma growth, with this effect being reversed at higher concentrations [220]. A separate study conducted on three glioblastoma cell lines (U251-MG, T98G, and CRT-MG) yielded similar findings. Natural antioxidants, such as quercetin, curcumin, tea polyphenols (e.g., EGCG), and vitamin D, have been shown to increase the activity of the Nrf2 pathway [221,222]. This pathway can target the transketolase gene, which connects the Pentose Phosphate Pathway (PPP) with glycolysis to affect the production of the antioxidant NADPH [223]. These mechanisms explain why antioxidants can sometimes promote tumor growth; they also pave the way for combination strategies aimed at precisely blocking undesirable protective effects. These findings suggest that the problem is not simply that antioxidants reduce ROS, but that they may actively reinforce the very pathways that cancer cells depend on for survival and metastasis. A natural extension of this observation is to ask whether these pro-oxidant effects can be blocked—and whether combining supplements with other agents might reverse or exploit this dynamic.

## 7. Inhibition of Pro-Oxidant Activities Through the Combined Use of Antioxidant Supplements and Other Molecules

Several experimental models illustrate this directly. In two colorectal cancer cell line experiments, NAC reversed ivermectin-induced ROS and cell death, but it promoted proliferation compared to the control group [224], thereby contributing to cancer progression. Similar results were observed in mouse and canine breast cancer models, where NAC reduced the ROS effects of a combination of ivermectin and metformin [225]. In non-small cell lung cancer, NAC inhibited mebendazole-induced ROS in two different cell lines [226]. Similar results are observed with vitamin C and NAC, in thyroid and breast cancer cell lines [30,207]. Vitamin C has been shown to exhibit pro-oxidant effects across 10 cancer cell lines (lung, breast, melanoma, pancreas, ovary, prostate, and cervical), with these effects being inhibited by glutathione. These results have been confirmed in xenografted mice [227]. 2-Deoxy-D-glucose (2-DG) suppresses doublecortin-like kinase 1, a cancer stem cell (CSC) pancreatic marker, which can be reversed by the combination of 2-DG + NAC, thus increasing cell viability [228]. Another study testing a series of drug combinations, including DCA, methylene blue (MB), and ALA, shows that ALA, although an antioxidant, can act as a pro-oxidant. However, in Figure 2 of the article, it can be observed that the combination of methylene blue and ALA, although pro-oxidant in this case, reduces the effects of MB on glutaminolysis [36], a noteworthy effect given that it is one of the two essential pathways for cancer cell survival [3,184,185,186]. Singh et al., 2017 [229] synthesized the results on the use of antioxidants in combination with chemotherapy. In humans, their findings indicate that the results of studies combining vitamins A, C, and E with standard chemotherapy are heterogeneous; some studies report beneficial effects on toxicity, therapeutic response, or survival, while others show no significant difference. Among non-vitamin antioxidants, melatonin shows consistently positive results across all evaluated outcomes, while GSH appears to primarily reduce treatment-related toxicity rather than affect survival. Antioxidant combinations seem to improve survival, particularly for combinations including vitamin A, vitamin E, and selenium, although the largest available study (n = 136) did not demonstrate any significant effect. A systematic review of RCTs including antioxidants such as glutathione, melatonin, vitamin A, antioxidant mixtures, vitamin C, NAC, vitamin E, and ellagic acid did not show a reduction in efficacy from antioxidant supplementation during chemotherapy, and even showed an improvement in survival for cancer patients [230]. Another systematic review shows that the total antioxidant status decreases during standard treatments for cancer patients [231]. Furthermore, the use of antioxidants (including vitamins A, C, and E; carotenoids; and coenzyme Q10) before and during chemotherapy has been linked to an increased risk of recurrence [232]. Similar results were observed during chemotherapy and after diagnosis for breast cancer [146]. This may be partly explained by the potential of standard treatments to fuel dormant tumor stem cells (see Section 9 of [185]). It is important to differentiate between the cytotoxicity induced by standard treatments such as chemotherapy, radiotherapy, and targeted therapies, which can be harmful to normal cells [233], and selective cytotoxicity directed towards cancer cells while preserving healthy cells. Indeed, in vitro studies have shown that vitamin C can kill cancer cells but not normal cells [234,235,236]. Other supplements have also demonstrated tumor-selective effects, without toxicity in normal cells, such as berberine in pancreatic and brain cancer cells [13,237,238], curcumin [239] and ALA [240] in colon cancer cells, α-tocopherol in oral cancer cells [241], and astaxanthin combined with β-carotene in breast cancer cell lines [242].

These results should be interpreted with caution, as most of the evidence comes from in vitro studies, and their reproducibility in vivo remains to be established. It may therefore be strategic to focus on supplements that exhibit selective pro-oxidant activity toward cancer cells while remaining non-toxic to healthy cells, with absorption and bioavailability (see Section 4) being key determinants of the effectiveness of oral supplementation. Taken together, these findings reveal the limits of evaluating supplements solely through their redox activity. Whether acting as antioxidants or pro-oxidants, their clinical impact cannot be fully explained by ROS modulation alone. This invites a broader look at the other biological mechanisms through which these compounds may influence tumor biology.

## 8. Alternative Mechanisms Potentially Surpassing the Antioxidant Effects of Supplements

Focusing exclusively on redox activity risks missing a broader and clinically relevant picture: several supplements exert potent anticancer effects through mechanisms that are largely independent of their antioxidant properties. While antioxidants have long been promoted for their protective roles against oxidative stress, a deeper examination of their broader biological activity reveals that their impact on health—particularly in the context of cancer—can be more complex. In fact, certain supplements may exert beneficial effects through mechanisms that surpass their antioxidant properties. These include their metabolic activity and their potential to influence the tumor microenvironment. Such alternative mechanisms could offer more sustainable and potent therapeutic benefits, especially in the context of cancer treatment, where tumor progression and resistance to standard therapies present ongoing challenges.

### 8.1. Metabolic Activity

Cancer cells, like all cells, require energy production. The majority of this energy is typically generated through oxidative phosphorylation. However, cancer cells exhibit mitochondrial alterations in number, structure, and function (see Table 1 [184]). These mitochondrial changes lead to a metabolic shift toward two essential pathways—glycolysis and mitochondrial substrate-level phosphorylation including glutaminolysis—which are inadequate for the survival of cancer cells [184,185,186]. The effects of supplements on these two pathways are summarized in Table 4 below.

ALA can also target two key enzymes in the metabolism of cancer cells, namely alpha-ketoglutarate dehydrogenase and pyruvate dehydrogenase [192]. An analog of ALA called CPI-613 (devimistat) has been developed and is currently undergoing trials [271]. Glucose serves as a precursor to the PPP for the creation of NADPH and glycine, supporting glutathione production [272]. Glutamine is converted to glutamate, which participates in the SLC7A11/Xc antiporter as well as directly contributing to the creation of glutathione [273]. Glutamine also serves as a way for the cell to create the NADPH required for all the endogenous redox systems to function. Many of these molecules can also regulate lipid metabolism. As a reminder, mitochondrial dysfunction in cancer cells leads to the expression of lipid droplets (which confirms mitochondrial alteration), which, in turn, are unable to produce ATP for cancer cells (see Table 2 [184]).

### 8.2. Other Mechanisms

Several additional mechanisms have been described for these supplements. Vitamin D, for example, can inhibit the renin–angiotensin system by reducing renin, which decreases angiotensin II levels and lowers angiotensin II receptor type 1 (AT1R) expression, with similar effects reported for EGCG and resveratrol [119,274]. These compounds, along with vitamin D, curcumin, berberine, and melatonin, have also been shown to suppress angiogenesis [119,275,276,277]. Immune-modulatory effects have also been observed: vitamin D and vitamin C enhance the function of both T cells (through epigenetic regulation) and NK cells, while melatonin regulates the tumor microenvironment and modulates immunity, particularly affecting NK and T cells, similar to selenium [99,119,277,278]. Vitamin D and vitamin C can further influence the microbiome, with vitamin D increasing Akkermansia and Bifidobacterium, and vitamin C promoting Bifidobacteria essential for maintaining gut eubiosis [119,279]. Finally, vitamin D, vitamin C, and ALA have been reported to target cancer stem cells [39,119,280]. These diverse mechanisms—metabolic, immunological, renin–angiotensin system-related, and microbiome-related—underscore the systemic reach of certain supplements.

## 9. Biomarkers for Monitoring Supplement Effects and Redox Activity

Translating these mechanistic insights into clinical practice requires validated biomarkers that can capture redox dynamics in real time. After identifying the alternative mechanisms that may surpass the simple antioxidant effects of supplements, it becomes essential to have concrete tools to monitor the true redox impact of these interventions in patients or clinical trials. Without appropriate monitoring, it remains difficult to distinguish whether a supplement exerts a protective, neutral, or paradoxically harmful effect, depending on the tumor context and the dose achieved.

Numerous validated biomarkers can thus be used to assess redox status and the effectiveness (or potential toxicity) of supplementation. The following are among the most relevant:

– Blood GSH/GSSG ratio [281]: a direct indicator of intracellular redox status.
– Plasma MDA (measured by HPLC) [282]: a marker of lipid peroxidation.
– Urinary 8-OHdG [283]: a specific marker of oxidative DNA damage.
– Plasma GPx3 activity [284]: as well as the antioxidant enzymes SOD and CAT, and regulatory pathways such as Nrf2 and the thioredoxin system.

The measurement of respiration-supported non-OxPhos pathways in humans could also be relevant for identifying the pathways used by the patient’s tumor, especially given the high intra-tumoral and inter-individual heterogeneity. Therefore, the measurement of these pathways during tumor biopsies using metabolomics, isotopic flux ^13^C, gene expression (RNA-seq), and protein expression (IHC) is a strategy that is likely to be relevant in the future. Non-invasive approaches exist—including the plasma measurement of circulating dihydroorotate/orotate, 5-ALA fluorescence-guided surgery, and PET choline—but they remain limited, indirect, or specific to a single pathway.

These biomarkers provide a dynamic view of redox balance and allow for the early detection of a potential pro-oxidant shift. The integration of these biomarkers into clinical practice or research protocols will enhance the understanding and optimization of supplement use in therapeutic contexts. With appropriate monitoring tools in place, the field is better positioned to design trials that move beyond empirical supplementation toward precision redox medicine. This shift in perspective is precisely what the future of integrative oncology requires.

## 10. Future Directions

The evidence reviewed here points toward a necessary reorientation: away from the reflexive use of antioxidants and toward a context-sensitive, dose-dependent, and mechanistically grounded framework. The relationship between antioxidant and pro-oxidant supplements and cancer is far from straightforward. While it is tempting to assume that reducing oxidative stress is universally beneficial, the evidence tells a more nuanced—and at times contradictory—story. In early or preventive contexts, antioxidant supplementation carries biological rationale; yet the clinical data remain stubbornly heterogeneous and largely disappointing (see Table 2). In advanced disease, where mitochondrial dysfunction predominates [3], the therapeutic logic inverts: exploiting the pro-oxidant potential of certain supplements to trigger apoptosis becomes the more compelling target. Nrf2 exemplifies this duality with striking clarity. As a gatekeeper of the antioxidant response, it can restrain progression toward high-grade lung malignancy [285]—yet its hyperactivation in primary murine cells paradoxically suppresses ROS sufficiently to promote tumorigenesis [286]. The same pathway, depending on context, can both suppress and fuel cancer. This complexity has concrete clinical consequences. In integrative medicine, antioxidant supplements are broadly valued and Nrf2 or Klotho stimulation is often considered inherently beneficial—an assumption that has generated dangerous misconceptions among practitioners and self-treating patients alike. Indiscriminate use of antioxidant supplements not only appears ineffective but may actively worsen recurrence and mortality. Paradoxically, certain supplements that are harmful at low oral doses demonstrate therapeutic benefit at high intravenous doses—as seen with ascorbic acid—suggesting that route and dose, not molecular identity alone, determine oncological outcomes. This dose-dependency points to a more fundamental principle: redox homeostasis, not antioxidant supplementation per se, is the true therapeutic target. Supplements exhibiting dual antioxidant/pro-oxidant activity are particularly promising, as pro-oxidant dosing may selectively overwhelm cancer cell defenses while remaining compatible with combination regimens. Rather than defaulting to antioxidant supplements, greater attention should be directed toward those with metabolic, microbiome-modulating, anti-angiogenic, or renin–angiotensin-targeting activity—mechanisms with more tractable therapeutic windows. When antioxidant supplements are being considered by health practitioners for cancer patients, we propose that four factors should be considered when interpreting the available evidence: (i) antioxidant effects should not predominate over anticancer effects; (ii) the absorption rate must be sufficient to potentially induce a pro-oxidant effect; (iii) as much as possible, verify that pro-oxidant doses are indeed being reached; and (iv) target the respiration-supported non-OxPhos pathways with specific supplements or therapeutic agents. Indeed, Chinopoulos emphasizes that therapeutic progress will come from interventions that collapse the respiratory redox infrastructure itself—particularly at the Q-junction and the CIII–cytochrome c–CIV segment—rather than strategies focused solely on depriving tumors of ATP. This perspective shifts the focus of anticancer supplements: it is not just about blocking ATP production, but disrupting mitochondrial redox states that support biosynthesis, signaling, and resistance to ferroptosis, independent of ATP synthase [189]. Numerous agents exist for almost all of the respiration-supported non-OxPhos pathways and could be used in combination (see Table 2 of [189]). Until such evidence is available, the use of antioxidant and pro-oxidant supplements in cancer care should be approached cautiously and selectively, focusing on those with proven benefits demonstrated in rigorous clinical studies.

Current RCT data remain insufficient to support definitive clinical recommendations, and these compounds should be prioritized in future well-designed, stratified clinical trials. Improving the bioavailability of other promising compounds, such as berberine, curcumin, and EGCG, also remains a path worth pursuing. Finally, this review has focused on the most commonly used supplements; it is likely that other compounds not examined here may also meet our four criteria and prove relevant for cancer patients, warranting systematic evaluation using the framework proposed.

## 11. Conclusions

A possible path forward may involve well-designed, stratified clinical trials to help clarify the safe and potentially effective pro-oxidant dosing of each supplement in the context of cancer care. Until more robust evidence becomes available, the use of antioxidant and pro-oxidant supplements in cancer patients should likely be approached with caution and considered selectively, prioritizing those for which benefits have been suggested by rigorous clinical studies. This cautious approach may help ensure that supplements contribute positively to patient outcomes while minimizing unintended risks and that they are integrated within a comprehensive, evidence-based therapeutic strategy.

## Figures and Tables

**Table 1:** Antioxidants with dual anti- and pro-oxidant activity in cancer cell lines.

Source	Cell Lines	Experimental Conditions	Supplement	Concentration	Exposure Time	Antioxidant	Pro-Oxidant
[11]	Breast (MCF-7 and MDA-MB-231)	Culture at 37 °C, appropriate medium (not specified) supplemented with 10% FBS, 1% non-essential amino acids, and 2% penicillin/streptomycin.	ALA	2 mM	48 h	ND	Increase intracellular ROS production
[12]	Liver (Huh-7 and Huh-7.HCVrep)	Culture at 37 °C, DMEM supplemented with 10% FBS, 1% gentamycin, 1% amphotericin B, 5% CO_2_.	Berberine	100 µM	24 h and 48 h	ND	Increase ROS production
[13]	Brain (DBTRG)	Culture at 37 °C, RPMI-1640 supplemented with 10% FBS, 1% penicillin/streptomycin, 1% L-glutamine, 5% CO_2_.	Berberine	0.5 to 10 μg/mL	48 h	Decrease ROS production	ND
[14,15]	Pancreas (MIA PaCa-2 and PANC1), retinoblastoma (Y79)	Culture at 37 °C, 5% CO_2_, in appropriate media (DMEM or RPMI). supplemented with 5–10% FBS and antibiotics	CoQ10	30 to 184.8 µM	24 h and 48 h	ND	Increase ROS production
[16,17,18]	Colon (HCT116), neuroblastoma (SK-N-SH) and cervical (SiHa)	Culture at 37 °C, 5% CO_2_ (20% O_2_ for HCT116), in McCoy’s 5A (HCT116) or DMEM (SiHa, SK-N-SH) supplemented with 10% FBS and penicillin/streptomycin.	Curcumin	8 to 50 µM	24 h and 48 h	ND	Increase ROS production
[19,20]	Oral (SCC25, SCC9, Tca8113, Cal27, and FaDu) and colon (HCT116)	Culture at 37 °C, 5% CO_2_, in DMEM (oral cancer cells, HCT116 uses RPMI) or M199 (HUVECs) supplemented with 10% FBS (20% for HUVECs), penicillin/streptomycin (+ heparin and bFGF for HUVECs).	Melatonin	1 mM	2 h and 24 h	Decrease ROS production	ND
[21]	Leukemia (HL-60 and U937)	Culture at 37 °C, 5% CO_2_, RPMI-1640 supplemented with 10% calf fetal serum and antibiotics.	NAC	0.25–4 mM	24 h	ND	Increase ROS production
[22]	Lung (A549),	Culture in high glucose DMEM supplemented with 10% FBS, 37 °C, 5% CO_2_.	NAC	16 µM to 5 mM	24 h, 72 h and 144 h	Decrease ROS production	ND
[23]	Cervical (HeLa)	Culture at 37 °C, 5% CO_2_, DMEM supplemented with 10% FBS.	Polyphenol (Resveratrol)	6.25 to 100 µM	30 min to 24 h	Decrease ROS production (30 min, 6.25 to 25 µM)	Increase ROS production (30 min, 50 to 100 µM; 24 h, 6.25 to 100 µM)
[24]	Colon (HCT116 and SW620)	Culture at 37 °C, 5% CO_2_, RPMI-1640 supplemented with 10% FBS.	Polyphenol (Resveratrol)	6 and 12 µg/mL	48 h	ND	Increase ROS production
[25]	Breast (MCF-7 and MDA-MB-231)	MCF-7: Eagle’s Minimum Essential Medium (EMEM) supplemented with 0.01 mg/mL human recombinant insulin, 1% NEAA, 1% sodium pyruvate, 10% FBS. MDA-MB-231: high glucose RPMI-1640 + 10% FBS, 2 mM L-glutamine, 1% penicillin-streptomycin-neomycin. Both at 37 °C, 5% CO_2_.	Polyphenol (Quercetin)	40 µM	30 min	ND	Increase ROS production
[26]	Breast (MCF-7)	Culture at 37 °C, 5% CO_2_, RPMI-1640 supplemented with 10% FBS, penicillin and streptomycin.	Polyphenol (Quercetin)	1 to 50 µM	30 min	Free radical scavenging	ND
[27]	Liver (HepG2)	Culture at 37 °C, 5% CO_2_, DMEM supplemented with 10% FBS and 1% penicillin-streptomycin.	Polyphenol (EGCG)	60 µM	6 h	ND	Increase ROS production
[28]	Cervical (HeLa, SiHa)	Culture at 37 °C, 5% CO_2_, RPMI-1640 supplemented with 10% FBS, 100 IU/mL penicillin and 100 µg/mL streptomycin.	Polyphenol (EGCG)	60 µM	24 h	Decrease ROS levels	ND
[29]	Lung (L9981)	Culture at 37 °C, 5% CO_2_, RPMI-1640 supplemented with 10% calf serum, 100 U/mL penicillin and 100 U/mL streptomycin.	Selenium	5 µM–5.5 µM	8 h and 12 h	ND	Increase ROS production
[30]	Thyroid (8305C, BCPAP, 8505C, FTC133, and TPC-1)	Culture at 37 °C, RPMI-1640 or DMEM/Ham’s F-12 supplemented with 10% FBS.	Vitamin C	2 mM	2 h and 4 h	ND	Increase ROS production
[31]	Breast (MDA-MB-231 and MDA-MB-468)	Culture at 37 °C, 5% CO_2_, DMEM supplemented with 10% FBS, 1% penicillin-streptomycin, GlutaMAX, NEAA, sodium pyruvate and 0.1% β-mercaptoethanol.	Vitamin C	10 and 20 mM	2 h	ND	Increase ROS production
[32]	Breast (MCF-7, MDA-MB-231), colon (HCT116, HT29), and head and neck (Detroit-562, FaDu)	All cells except FaDu: RPMI-1640 supplemented with 10% FBS, 1% sodium pyruvate and 1% L-glutamine. FaDu: MEM supplemented with 10% FBS, 1% sodium pyruvate, 1% L-glutamine and 1% NEAA.	Vitamin D	1 to 100 nM	48 h	Decrease ROS production	ND
[33]	Breast (MCF-7)	Culture at 37 °C, 5% CO_2_, RPMI-1640 supplemented with 10% FBS, 100 U/mL penicillin and 100 µg/mL streptomycin.	Vitamin E (Trolox)	100 µM	24 h	Decrease ROS levels	ND
[34]	Prostate (PC-3, DU145)	Culture at 37 °C, 5% CO_2_/95% air, RPMI supplemented with glutamine, antibiotics and FBS (7.5% for PC3, 5% for DU145).	Vitamin E	15 µg/ml	12 h	ND	δ-tocotrienol increases ROS production
[35]	Breast (MDA-MB-231, SUM159)	MDA-MB-231: MEM + 10% FBS; SUM159: Ham’s F12 + 5% FBS, 5 µg/mL insulin, 1 µg/mL hydrocortisone, 10 mM HEPES.	Vitamin E	5 to 20 µM	3 h	α-tocopherol decreased ROS levels (20 µM)	ND

ALA: Alpha-Lipoic Acid; CoQ10: Coenzyme Q10; EGCG: Epigallocatechin Gallate; mM: milliMolar; NAC: N-Acetylcysteine; ND: Not Determined; ROS: Reactive Oxygen Species.

**Table 3:** Effects of supplements on respiration-supported non-OxPhos mitochondrial pathways.

Supplement	Respiration-Supported Non-OxPhos Pathway	Outcomes Related to Chinopoulos’ Pathway Description [189]
ALA	↑ UQH_2_ => ↓ DHODH [190], supports mitochondrial lipoylation (PDH, α-KGDH, GCS) [191], ↑ TCA cycle [192];	↓ (i) and ↑ (vi)
Berberine	↓ complex I => ↓ GOT2 => ↓ aspartate [193,194], ↓ TCA cycle [195];	↓ (ii) and ↓ (vi)
CoQ10	↑ de novo pyrimidine synthesis via DHODH [196], ↑ SQOR, ↑ H_2_S oxidation [197], ↑ NNT-GSH [198];	↑ (i), (iv), (vii), and (ix).
Curcumin	↓ TCA cycle [199];	↓ (vi)
EGCG	↓ TCA cycle via GLUD inhibition [200,201].	↓ (vi)
Melatonin	↑ Proline, ↑ TCA cycle [202];	↑ (vi), (viii)
NAC	↑ H_2_S → SQOR → ↑ UQH_2_ → ↓ ferroptosis [203], indirect effect on NNT [204];	↑ (ix)
Quercetin	No reported direct interaction with respiration-supported non-OxPhos pathways in the literature reviewed;	ND
Resveratrol	↑ TCA cycle [205];	↑ (vi)
Selenium	↑ H_2_Se → SQOR → ↑ UQH_2_ → ↓ ferroptosis [206];	↑ (ix)
Vitamin C	↑ Fe^3+^ to Fe^2+^ => ↑ ferroptosis [107], ↓ TCA cycle [207,208];	↓ (vi) and ↓ (vii)
Vitamin D	Redirects TCA cycle metabolites [209], ↓ TCA cycle [210];	↓ (vi)
Vitamin E	↓ ferroptosis [211]; ↑ TCA cycle [210].	↑ (vi), ↑ (vii)

The directional arrows represent system-level modulation of respiration-supported non-OxPhos mitochondrial pathways, integrating evidence from multiple mechanistic studies. α-KGDH: Alpha-Ketoglutarate Dehydrogenase; ALA: Alpha-Lipoic Acid; CoQ10: Coenzyme Q10; DHODH: Dihydroorotate Dehydrogenase; EGCG: Epigallocatechin Gallate; Fe-S: Iron-Sulfur cluster; GCS: Glycine Cleavage System; GLUD: Glutamate Dehydrogenase; GOT2: Glutamate–Oxaloacetate Transaminase 2; GSH: Glutathione; H_2_S: Hydrogen Sulfide; H_2_Se: Hydrogen Selenide; NAC: N-Acetylcysteine; ND: Not Determined; NNT: Nicotinamide Nucleotide Transhydrogenase; PDH: Pyruvate Dehydrogenase; SQOR: Sulfide: Quinone Oxidoreductase; TCA cycle: Tricarboxylic Acid cycle (Krebs cycle); UQH_2_: Ubiquinol.

**Table 4:** Summary of metabolic activity of supplements.

Source	Cell Lines	Supplement	Metabolism
[40]	Breast (MCF-7 and MDA-MB-231)	ALA	Decreased glycolysis by downregulating the expression of PKM2 and LDHA.
[243]	Colon (HCT116 and KM12C)	Berberine	Decreased glycolysis metabolism via suppression of mTOR-dependent HIF-1α protein.
[244]	Ovarian (SKOV3 and HEY)	Berberine	Decreased glycolysis through the LINC01123/P65/MAPK10 signaling axis.
[47]	Gastrointestinal (CT26, HT29, TMK1)	Berberine	Increased glycolysis and glutaminolysis (pyruvate kinase M, enzyme-1, acetyl-CoA carboxylase α, and glucose-6-phosphate dehydrogenase).
[245]	Liver (Hep3B and BEL-7404)	Berberine	Decreased glutaminolysis by inhibiting glutamine transporter (SLC1A5).
[246]	Lung (H1299 and A549)	Berberine	Berberine inhibits the MT-CO2-GLS1 axis to decrease the glutaminolysis pathway.
[247]	Brain (T67)	Coenzyme Q10	Decreased glycolysis through HIF-1a.
[248,249]	Colon (HCT116 and HT29), prostate (PC-3AcT and DU145AcT)	Curcumin	Decreased glycolysis via HK-II.
[250]	Lung (H1299), breast (MCF-7), cervical (HeLa), and prostate (PC-3)	Curcumin	Decreased glycolysis via by downregulating PKM2 expression, via inhibition of the mTOR-HIF1α axis.
[251]	Colon (HT29)	Curcumin	Decreased glutaminolysis via MicroRNA-137–glutaminase axis.
[252]	Cholangiocarcinoma (KKU-213B and KKU-213BGemR)	Curcumin	Decreased glutaminolysis via inhibiting LAT2, GLS, and GS pathway.
[253]	Bladder (T24, 5637, UM-UC3)	Melatonin	Decreased glycolysis by silencing the ENO1 upstream factor PPARγ.
[57,58]	Lung (A549), pancreas (PANC-1), prostate (DU145, PC-3, and LNCaP) and cervical (Hela)	Melatonin	Direct inhibitor of HIF-1α.
[254]	Squamous cell carcinoma of the head and neck (Cal-27 and SCC9)	Melatonin	Decreased glycolysis through HK-II.
[255]	Breast (BT549 and MDA-MB-231)	Melatonin	Decreased glycolysis by inhibition of YAP1 signaling.
[256]	Ovarian (SKOV3 and CAISMOV-24)	Melatonin	Decreased glycolysis and glutaminolysis through PFK-1, G6PDH, LDH, CS, and GS.
[257]	Pancreatic (PANC-1 and AsPC-1)	Melatonin	Decreased glutaminolysis through PERK-eIF2α-ATF4 axis.
[258]	Prostate (LNCaP)	Melatonin	Decreased GLUT1 receptor.
[259]	Breast and liver (MCF-7 and HepG2)	Resveratrol	Decreased glycolysis by downregulating the expression of PKM2.
[260]	Lung (H460 and HCC827)	Resveratrol	Decreased glycolysis by inhibition of HK-II.
[261]	Ovarian (A2780, OVCA429, CADV3, and SKOV3)	Resveratrol	Decreased glycolysis via AKT and mTOR pathways.
[262]	Liver (C3A and SMCC7721)	Resveratrol	Decreased glutaminolysis by inhibition of glutamine membrane transporter ASCT2.
[263]	Breast (MCF-7 and MDA-MB-231)	Polyphenols (quercetin and EGCG)	Decreased glycolysis by reducing GLUT1 receptor.
[201]	Colon (HCT116)	Polyphenols (EGCG)	Decreased glutaminolyse by inhibition of GLUD
[264]	Neuroblastoma (IMR32, SH-SY5Y, DF2 and FRSN)	Polyphenols (curcumin, quercetin, and resveratrol and combinations)	Decreased glycolysis and glutaminolysis by inhibition of HK-II, PFKM, PKM2, LDHA, and GLS1.
[265,266]	Lung (A549) and colon (HCT116 and HT29)	Selenium (selenite)	Decreased glutaminolysis by inhibition of GLS1.
[207]	Breast (MCF-7) and colon (HT29)	Vitamin C	Decreased glycolysis and PPP pathways and TCA cycle.
[108]	Melanoma (WM1366)	Vitamin C	Decreased HIF-1α.
[208]	Breast (MCF-7) and prostate (PC-3)	Vitamin C	Decreased glutaminolysis pathway by inhibition of glutamine synthetase.
[267]	Breast (MCF-7 and MDA-MB-231)	Vitamin D	Decreased glycolysis through HK-II, in MCF-7, but increased HK-II in MDA-MB-231 cells and decreased glutaminolysis pathway.
[268]	Breast (MCF-7 and MDA-MB-231)	Vitamin D	Decreased glycolysis through GLUT1, HK-II, and LDHA
[269]	Breast (Harvey-ras transformed MCF10A)	Vitamin D	Decreased glutaminolysis pathway through downregulation of SLC1A5.
[270]	Breast (MCF-7 and +SA)	Vitamin E	Decreased glycolysis through HK-II, phosphofructokinase, PKM2, and LDHA.

AKT: Protein Kinase B; ASCT2: Alanine Serine Cysteine Transporter 2; ENO1: Enolase 1; G6PDH: Glucose-6-Phosphate Dehydrogenase; GLS/GLS1: Glutaminase/Glutaminase 1; GLUD: Glutamate Dehydrogenase; GLUT1: Glucose Transporter 1; GS: Glutamine Synthetase; HIF-1α: Hypoxia-Inducible Factor 1-alpha; HK-II: Hexokinase II; LAT2: L-type amino-acid transporter-2; LDH: Lactate Dehydrogenase; MAPK10: Mitogen-Activated Protein Kinase 10; miR-137: MicroRNA-137; mTOR: Mechanistic Target Of Rapamycin; PERK: Protein Kinase R-like Endoplasmic Reticulum Kinase; PFK-1: Phosphofructokinase 1; PFKM: Phosphofructokinase Muscle-type; PKM/PKM2: Pyruvate Kinase M/Pyruvate Kinase M2; PPARγ: Peroxisome Proliferator-Activated Receptor Gamma; PPP: Pentose Phosphate Pathway; SLC1A5; Solute Carrier Family 1 Member 5; TCA cycle: Tricarboxylic Acid Cycle; YAP1: Yes-Associated Protein 1.

## Data Availability

No new data were created or analyzed in this study. Data sharing is not applicable to this article.

## Abbreviations

2-DG	2-Deoxy-D-Glucose
ALA	α-Lipoic Acid
ASCT2	Alanine Serine Cysteine Transporter 2—glutamine membrane transporter
ASK	Apoptosis Signaling Kinase
ATP	Adenosine Triphosphate
ATF4	Activating Transcription Factor 4
AUC	Area Under the Curve
BACH1	Basic Leucine Heme-regulated Transcription Factor 1
BBR	Berberine
Bcl-2	B-Cell Lymphoma 2 protein family
BMI	Body Mass Index
CAT	Catalase
CoQ10	Coenzyme Q10 (ubiquinone/ubiquinol)
CSC	Cancer Stem Cell
Cyt-c	Cytochrome c
DCA	Dichloroacetic Acid
DHODH	Dihydroorotate Dehydrogenase
EGCG	Epigallocatechin Gallate
EMT	Epithelial–Mesenchymal Transition
ENO1	Enolase enzyme
ETC	Electron Transport Chain
G6PDH	Glucose-6-Phosphate Dehydrogenase
GAPDH	Glyceraldehyde-3-Phosphate Dehydrogenase
GLS/GLS1	Glutaminase/Glutaminase 1
GLUD	Glutamate Dehydrogenase
GSH	Glutathione
GSSG	Oxidized Glutathione
GS	Glutamine Synthetase
GCLC	Glutamate–Cysteine Ligase Catalytic subunit
GCLM	Glutamate–Cysteine Ligase Modifier subunit
GCS	Glycine Cleavage System
GOT2	Glutamic–Oxaloacetic Transaminase, a pyridoxal phosphate-dependent enzyme present in cytoplasmic and mitochondrial forms
GPx	Glutathione Peroxidase
GR	Glutathione Reductase
HIF	Hypoxia-Inducible Factor
HK-II	Hexokinase II
HMG-CoA	3-Hydroxy-3-Methylglutaryl Coenzyme A
HO-1	Heme Oxygenase
JAK	Janus Tyrosine Kinase
JNK	c-Jun N-terminal Kinase
K-Ras	Kirsten Rat Sarcoma, gene and protein
Keap1	Kelch-like ECH-associated protein 1
LAT2	L-type Amino Acid Transporter 2
LDHA	Lactate Dehydrogenase A
LDH	Lactate Dehydrogenase
MDA	Malondialdehyde
MB	Methylene Blue
mPTP	Mitochondrial Permeability Transition Pore
mSLP	Mitochondrial Substrate-Level Phosphorylation
mTOR	Mechanistic Target of Rapamycin
NAC	N-Acetylcysteine
NADPH	Nicotinamide Adenine Dinucleotide Phosphate Hydrogen
NK cells	Natural Killer cells
NNT	Nicotinamide Nucleotide Transhydrogenase
NQO1	NAD(P)H Quinone Oxidoreductase 1
Nrf2	Nuclear Factor Erythroid 2–related Factor 2, transcription factor
NOX/NOX2/NOX4	NADPH Oxidase (isoforms 2 and 4)
NRP	Plasma membrane-associated Neuropilin
NSCLC	Non-Small Cell Lung Cancer
OxPhos	Oxidative Phosphorylation
PGC-1α	Peroxisome Proliferator-Activated Receptor Gamma Coactivator 1-alpha
PERK	Protein kinase R-like Endoplasmic Reticulum Kinase
PFK-1	Phosphofructokinase 1
PFKM	Phosphofructokinase Muscle type
P-gp	P-glycoprotein
PKM2	Pyruvate Kinase Muscle Isoform 2
PPAR	Peroxisome Proliferator-Activated Receptor
PPP	Pentose Phosphate Pathway
PRDX5	Peroxiredoxin 5
Ras-Raf-MEK-ERK	Protein signaling cascade; ERK = Extracellular Signal-Regulated Kinase
RCT	Randomized Controlled Trial
ROS	Reactive Oxygen Species
SIRT1	Sirtuin 1
SLC1A5	Solute Carrier Family 1 Member 5
SLC7A11	Solute Carrier Family 7 Member 11 (xCT, cystine/glutamate antiporter)
SOD	Superoxide Dismutase enzyme
SQOR	Sulfide:Quinone Oxidoreductase
STAT	Signal Transducer and Activator of Transcription
SVCT1/2	Sodium-dependent Vitamin C Transporter 1/2
TCA	Tricarboxylic Acid cycle
Trx	Thioredoxin
TrxR	Thioredoxin Reductase
UQH_2_	Ubiquinol (reduced form of ubiquinone)
VEGF	Vascular Endothelial Growth Factor
VDR	Vitamin D Receptor
YAP1	Yes-Associated Protein 1

## References

[1] Jee S.-C., Cheong H. (2023). Autophagy/Mitophagy Regulated by Ubiquitination: A Promising Pathway in Cancer Therapeutics. Cancers.

[2] Akter S., Madhuvilakku R., Kar A.K., Nila I.S., Liu P., Inuzuka H., Wei W., Hong Y. (2026). Reactive Oxygen Species (ROS) in Cancer: From Mechanism to Therapeutic Implications. Signal Transduct. Target. Ther..

[3] Seyfried T.N., Arismendi-Morillo G., Mukherjee P., Chinopoulos C. (2020). On the Origin of ATP Synthesis in Cancer. iScience.

[4] Nakamura H., Takada K. (2021). Reactive Oxygen Species in Cancer: Current Findings and Future Directions. Cancer Sci..

[5] Schmidt S., Qiao X., Bergö M.O. (2025). Effects of Antioxidants on Cancer Progression. EMBO Mol. Med..

[6] Lesperance M.L., Olivotto I.A., Forde N., Zhao Y., Speers C., Foster H., Tsao M., MacPherson N., Hoffer A. (2002). Mega-Dose Vitamins and Minerals in the Treatment of Non-Metastatic Breast Cancer: An Historical Cohort Study. Breast Cancer Res. Treat..

[7] Du M., Luo H., Blumberg J.B., Rogers G., Chen F., Ruan M., Shan Z., Biever E., Zhang F.F. (2020). Dietary Supplement Use among Adult Cancer Survivors in the United States. J. Nutr..

[8] Kristoffersen A.E., Stub T., Nilsen J.V., Nordberg J.H., Broderstad A.R., Wider B., Bjelland M. (2024). Exploring Dietary Changes and Supplement Use among Cancer Patients in Norway: Prevalence, Motivations, Disclosure, Information, and Perceived Risks and Benefits: A Cross Sectional Study. BMC Nutr..

[9] Halma M.T.J., Tuszynski J.A., Marik P.E. (2023). Cancer Metabolism as a Therapeutic Target and Review of Interventions. Nutrients.

[10] Pulliero A., Marengo B., Ferrante O., Khalid Z., Vernazza S., Ruzzarin N., Domenicotti C., Izzotti A. (2025). Antioxidant Food Supplementation in Cancer: Lessons from Clinical Trials and Insights from Preclinical Studies. Antioxidants.

[11] Farhat D., Ghayad S.E., Icard P., Le Romancer M., Hussein N., Lincet H. (2020). Lipoic Acid-Induced Oxidative Stress Abrogates IGF-1R Maturation by Inhibiting the CREB/Furin Axis in Breast Cancer Cell Lines. Oncogene.

[12] Tai C.-J., Jassey A., Liu C.-H., Tai C.-J., Richardson C.D., Wong S.H., Lin L.-T. (2020). Targeting Autophagy Augments Berberine-Mediated Cell Death in Human Hepatoma Cells Harboring Hepatitis C Virus RNA. Cells.

[13] Shen C.-C., Yang M.-Y., Hsieh W.-Y., Tsay G.J., Yang Y.-C., Huang Y.-F., Liu S.-Y., Lai C.-M., Lee C.H., Tang C.-M. (2025). Berberine’s Impact on Apoptosis, Proliferation, Uptake Efficiency, and Nanoparticle-Based Therapy in DBTRG Cells. ACS Nanosci. Au.

[14] Dadali T., Diers A.R., Kazerounian S., Muthuswamy S.K., Awate P., Ng R., Mogre S., Spencer C., Krumova K., Rockwell H.E. (2021). Elevated Levels of Mitochondrial CoQ10 Induce ROS-Mediated Apoptosis in Pancreatic Cancer. Sci. Rep..

[15] Upreti S., Sharma P., Sen S., Biswas S., Ghosh M.P. (2024). Auxiliary Effect of Trolox on Coenzyme Q10 Restricts Angiogenesis and Proliferation of Retinoblastoma Cells via the ERK/Akt Pathway. Sci. Rep..

[16] Liu C., Rokavec M., Huang Z., Hermeking H. (2023). Curcumin Activates a ROS/KEAP1/NRF2/miR-34a/b/c Cascade to Suppress Colorectal Cancer Metastasis. Cell Death Differ..

[17] Wang T., Wu X., Al Rudaisat M., Song Y., Cheng H. (2020). Curcumin Induces G2/M Arrest and Triggers Autophagy, ROS Generation and Cell Senescence in Cervical Cancer Cells. J. Cancer.

[18] Ye Z., Chen D., Zheng R., Chen H., Xu T., Wang C., Zhu S., Gao X., Zhang J., Li D. (2021). Curcumin Induced G2/M Cycle Arrest in SK-N-SH Neuroblastoma Cells through the ROS-mediated P53 Signaling Pathway. J. Food Biochem..

[19] Liu R., Wang H., Deng M., Wen X., Mo Y., Chen F., Zou C., Duan W., Li L., Nie X. (2018). Melatonin Inhibits Reactive Oxygen Species-Driven Proliferation, Epithelial-Mesenchymal Transition, and Vasculogenic Mimicry in Oral Cancer. Oxid. Med. Cell. Longev..

[20] Park S.-Y., Jang W.-J., Yi E.-Y., Jang J.-Y., Jung Y., Jeong J.-W., Kim Y.-J. (2010). Melatonin Suppresses Tumor Angiogenesis by Inhibiting HIF-1α Stabilization under Hypoxia. J. Pineal Res..

[21] Mlejnek P., Dolezel P., Kriegova E., Pastvova N. (2021). N-Acetylcysteine Can Induce Massive Oxidative Stress, Resulting in Cell Death with Apoptotic Features in Human Leukemia Cells. Int. J. Mol. Sci..

[22] Montero P., Roger I., Estornut C., Milara J., Cortijo J. (2023). Influence of Dose and Exposition Time in the Effectiveness of N-Acetyl-l-Cysteine Treatment in A549 Human Epithelial Cells. Heliyon.

[23] Zhang Y., Yuan F., Li P., Gu J., Han J., Ni Z., Liu F. (2022). Resveratrol Inhibits HeLa Cell Proliferation by Regulating Mitochondrial Function. Ecotoxicol. Environ. Saf..

[24] Fu Y., Ye Y., Zhu G., Xu Y., Sun J., Wu H., Feng F., Wen Z., Jiang S., Li Y. (2020). Resveratrol Induces Human Colorectal Cancer Cell Apoptosis by Activating the Mitochondrial Pathway via Increasing Reactive Oxygen Species. Mol. Med. Rep..

[25] Sakao K., Hamamoto S., Urakawa D., He Z., Hou D.-X. (2024). Anticancer Activity and Molecular Mechanisms of Acetylated and Methylated Quercetin in Human Breast Cancer Cells. Molecules.

[26] Rezaei-Sadabady R., Eidi A., Zarghami N., Barzegar A. (2016). Intracellular ROS Protection Efficiency and Free Radical-Scavenging Activity of Quercetin and Quercetin-Encapsulated Liposomes. Artif. Cells Nanomed. Biotechnol..

[27] Zhang Y., Yang N.-D., Zhou F., Shen T., Duan T., Zhou J., Shi Y., Zhu X.-Q., Shen H.-M. (2012). (−)-Epigallocatechin-3-Gallate Induces Non-Apoptotic Cell Death in Human Cancer Cells via ROS-Mediated Lysosomal Membrane Permeabilization. PLoS ONE.

[28] Panji M., Behmard V., Zare Z., Malekpour M., Nejadbiglari H., Yavari S., Nayerpour Dizaj T., Safaeian A., Maleki N., Abbasi M. (2021). Suppressing Effects of Green Tea Extract and Epigallocatechin-3-Gallate (EGCG) on TGF-β- Induced Epithelial-to-Mesenchymal Transition via ROS/Smad Signaling in Human Cervical Cancer Cells. Gene.

[29] Liu C., Chen J., Wu Z., Li Y., Zhu Y., Ren Y., Zhou Q. (2008). Selenium compounds induce ROS in human high-metastatic large cell lung cancer cell line L9981. Zhongguo Fei Ai Za Zhi Chin. J. Lung Cancer.

[30] Su X., Shen Z., Yang Q., Sui F., Pu J., Ma J., Ma S., Yao D., Ji M., Hou P. (2019). Vitamin C Kills Thyroid Cancer Cells through ROS-Dependent Inhibition of MAPK/ERK and PI3K/AKT Pathways via Distinct Mechanisms. Theranostics.

[31] Sen U., Chaudhury D., Shenoy P.S., Bose B. (2021). Differential Sensitivities of Triple-negative Breast Cancer Stem Cell towards Various Doses of Vitamin C: An Insight into the Internal Antioxidant Systems. J. Cell. Biochem..

[32] Elsalem L., Shobaki F.A., Al-Azzam N., Aleikish A.A., Basheer H.A. (2026). In Vitro Investigations on the Antioxidant Effects of Vitamin D in a Panel of Cancer Cell Lines. Biomedicines.

[33] Diao Q.X., Zhang J.Z., Zhao T., Xue F., Gao F., Ma S.M., Wang Y. (2016). Vitamin E Promotes Breast Cancer Cell Proliferation by Reducing ROS Production and P53 Expression. Eur. Rev. Med. Pharmacol. Sci..

[34] Fontana F., Raimondi M., Marzagalli M., Audano M., Beretta G., Procacci P., Sartori P., Mitro N., Limonta P. (2020). Mitochondrial Functional and Structural Impairment Is Involved in the Antitumor Activity of δ-Tocotrienol in Prostate Cancer Cells. Free Radic. Biol. Med..

[35] Xiong A., Yu W., Tiwary R., Sanders B.G., Kline K. (2012). Distinct Roles of Different Forms of Vitamin E in DHA-induced Apoptosis in Triple-negative Breast Cancer Cells. Mol. Nutr. Food Res..

[36] Montégut L., Martínez-Basilio P.C., Da Veiga Moreira J., Schwartz L., Jolicoeur M. (2020). Combining Lipoic Acid to Methylene Blue Reduces the Warburg Effect in CHO Cells: From TCA Cycle Activation to Enhancing Monoclonal Antibody Production. PLoS ONE.

[37] Hecht F., Zocchi M., Alimohammadi F., Harris I.S. (2024). Regulation of Antioxidants in Cancer. Mol. Cell.

[38] Cronan J.E. (2020). Progress in the Enzymology of the Mitochondrial Diseases of Lipoic Acid Requiring Enzymes. Front. Genet..

[39] Yan S., Lu J., Chen B., Yuan L., Chen L., Ju L., Cai W., Wu J. (2024). The Multifaceted Role of Alpha-Lipoic Acid in Cancer Prevention, Occurrence, and Treatment. Antioxidants.

[40] Chakravarti B., Tomar M.S., Qais F.A., Raza S., Abdullah K.M., Sharma G., Tewari A., Yadav A., Gupta P., Chattopadhyay N. (2025). Alpha Lipoic Acid Modulates Metabolic Reprogramming in Breast Cancer Stem Cells Enriched 3D Spheroids by Targeting Phosphoinositide 3-Kinase: In Silico and in Vitro Insights. Biomed. Pharmacother..

[41] Steele M.L., Fuller S., Patel M., Kersaitis C., Ooi L., Münch G. (2013). Effect of Nrf2 Activators on Release of Glutathione, Cysteinylglycine and Homocysteine by Human U373 Astroglial Cells. Redox Biol..

[42] Palaniappan A.R., Dai A. (2007). Mitochondrial Ageing and the Beneficial Role of α-Lipoic Acid. Neurochem. Res..

[43] Guo Y., Jones D., Palmer J.L., Forman A., Dakhil S.R., Velasco M.R., Weiss M., Gilman P., Mills G.M., Noga S.J. (2014). Oral Alpha-Lipoic Acid to Prevent Chemotherapy-Induced Peripheral Neuropathy: A Randomized, Double-Blind, Placebo-Controlled Trial. Support. Care Cancer.

[44] Werida R.H., Elshafiey R.A., Ghoneim A., Elzawawy S., Mostafa T.M. (2022). Role of Alpha-Lipoic Acid in Counteracting Paclitaxel- and Doxorubicin-Induced Toxicities: A Randomized Controlled Trial in Breast Cancer Patients. Support. Care Cancer.

[45] Hsu C., Pallathadka H., Gupta J., Ma H., Al-Shukri H.H.K., Kareem A.K., Zwamel A.H., Mustafa Y.F. (2024). Berberine and Berberine Nanoformulations in Cancer Therapy: Focusing on Lung Cancer. Phytother. Res..

[46] Rauf A., Abu-Izneid T., Khalil A.A., Imran M., Shah Z.A., Emran T.B., Mitra S., Khan Z., Alhumaydhi F.A., Aljohani A.S.M. (2021). Berberine as a Potential Anticancer Agent: A Comprehensive Review. Molecules.

[47] Mori S., Fujiwara-Tani R., Gyoten M., Nukaga S., Sasaki R., Ikemoto A., Ogata R., Kishi S., Fujii K., Kuniyasu H. (2023). Berberine Induces Combined Cell Death in Gastrointestinal Cell Lines. Int. J. Mol. Sci..

[48] Chen Q., Hou Y., Li D., Ding Z., Xu X., Hao B., Xia Q., Li M., Fan L. (2022). Berberine Induces Non-Small Cell Lung Cancer Apoptosis via the Activation of the ROS/ASK1/JNK Pathway. Ann. Transl. Med..

[49] Ghasempour Dabaghi G., Rabiee Rad M., Mohammad-Zamani M., Karimi Shervedani A., Bahrami-Samani F., Heshmat-Ghahdarijani K. (2024). The Role of Coenzyme Q10 as a Preventive and Therapeutic Agent for the Treatment of Cancers. Curr. Probl. Cancer.

[50] Wang Y., Lilienfeldt N., Hekimi S. (2024). Understanding Coenzyme Q. Physiol. Rev..

[51] Samimi F., Baazm M., Nadi Z., Dastghaib S., Rezaei M., Jalali-Mashayekhi F. (2024). Evaluation of Antioxidant Effects of Coenzyme Q10 against Hyperglycemia-Mediated Oxidative Stress by Focusing on Nrf2/Keap1/HO-1 Signaling Pathway in the Liver of Diabetic Rats. Iran. J. Med. Sci..

[52] Chai W., Cooney R.V., Franke A.A., Shvetsov Y.B., Caberto C.P., Wilkens L.R., Le Marchand L., Henderson B.E., Kolonel L.N., Goodman M.T. (2010). Plasma Coenzyme Q10 Levels and Postmenopausal Breast Cancer Risk: The Multiethnic Cohort Study. Cancer Epidemiol. Biomark. Prev..

[53] Li Y., Li S., Zhou Y., Meng X., Zhang J.-J., Xu D.-P., Li H.-B. (2017). Melatonin for the Prevention and Treatment of Cancer. Oncotarget.

[54] Guo Y., Sun J., Li T., Zhang Q., Bu S., Wang Q., Lai D. (2017). Melatonin Ameliorates Restraint Stress-Induced Oxidative Stress and Apoptosis in Testicular Cells via NF-κB/iNOS and Nrf2/HO-1 Signaling Pathway. Sci. Rep..

[55] Guo P., Pi H., Xu S., Zhang L., Li Y., Li M., Cao Z., Tian L., Xie J., Li R. (2014). Melatonin Improves Mitochondrial Function by Promoting MT1/SIRT1/PGC-1 Alpha-Dependent Mitochondrial Biogenesis in Cadmium-Induced Hepatotoxicity In Vitro. Toxicol. Sci..

[56] Proietti S., Cucina A., Minini M., Bizzarri M. (2017). Melatonin, Mitochondria, and the Cancer Cell. Cell. Mol. Life Sci..

[57] Dai M., Cui P., Yu M., Han J., Li H., Xiu R. (2008). Melatonin Modulates the Expression of VEGF and HIF-1α Induced by CoCl_2_ in Cultured Cancer Cells. J. Pineal Res..

[58] Park J., Hwang M., Suh S., Baek W. (2009). Melatonin Down-regulates HIF-1α Expression through Inhibition of Protein Translation in Prostate Cancer Cells. J. Pineal Res..

[59] Lissoni P. (2007). Biochemotherapy with Standard Chemotherapies plus the Pineal Hormone Melatonin in the Treatment of Advanced Solid Neoplasms. Pathol. Biol..

[60] Lissoni P. (2007). Biochemotherapy with Immunomodulating Pineal Hormones Other than Melatonin: 5-Methoxytryptamine as a New Oncostatic Pineal Agent. Pathol. Biol..

[61] Lissoni P., Barni S., Mandalà M., Ardizzoia A., Paolorossi F., Vaghi M., Longarini R., Malugani F., Tancini G. (1999). Decreased Toxicity and Increased Efficacy of Cancer Chemotherapy Using the Pineal Hormone Melatonin in Metastatic Solid Tumour Patients with Poor Clinical Status. Eur. J. Cancer.

[62] Lissoni P., Chilelli M., Villa S., Cerizza L., Tancini G. (2003). Five Years Survival in Metastatic Non-small Cell Lung Cancer Patients Treated with Chemotherapy Alone or Chemotherapy and Melatonin: A Randomized Trial. J. Pineal Res..

[63] Cerea G., Vaghi M., Ardizzoia A., Villa S., Bucovec R., Mengo S., Gardani G., Tancini G., Lissoni P. (2003). Biomodulation of Cancer Chemotherapy for Metastatic Colorectal Cancer: A Randomized Study of Weekly Low-Dose Irinotecan Alone versus Irinotecan plus the Oncostatic Pineal Hormone Melatonin in Metastatic Colorectal Cancer Patients Progressing on 5-Fluorouracil-Containing Combinations. Anticancer. Res..

[64] Lissoni P., Paolorossi F., Ardizzoia A., Barni S., Chilelli M., Mancuso M., Tancini G., Conti A., Maestroni G.J.M. (1997). A Randomized Study of Chemotherapy with Cisplatin plus Etoposide versus Chemoendocrine Therapy with Cisplatin, Etoposide and the Pineal Hormone Melatonin as a First-line Treatment of Advanced Non-small Cell Lung Cancer Patients in a Poor Clinical State. J. Pineal Res..

[65] Lissoni P., Tancini G., Barni S., Paolorossi F., Ardizzoia A., Conti A., Maestroni G. (1997). Treatment of Cancer Chemotherapy-Induced Toxicity with the Pineal Hormone Melatonin. Support. Care Cancer.

[66] Lim S., Park S., Koyanagi A., Yang J.W., Jacob L., Yon D.K., Lee S.W., Kim M.S., Il Shin J., Smith L. (2022). Effects of Exogenous Melatonin Supplementation on Health Outcomes: An Umbrella Review of Meta-Analyses Based on Randomized Controlled Trials. Pharmacol. Res..

[67] Mills E., Wu P., Seely D., Guyatt G. (2005). Melatonin in the Treatment of Cancer: A Systematic Review of Randomized Controlled Trials and Meta-analysis. J. Pineal Res..

[68] Seely D., Wu P., Fritz H., Kennedy D.A., Tsui T., Seely A.J.E., Mills E. (2012). Melatonin as Adjuvant Cancer Care with and Without Chemotherapy: A Systematic Review and Meta-Analysis of Randomized Trials. Integr. Cancer Ther..

[69] Veiga E.C.D.A., Simões R., Valenti V.E., Cipolla-Neto J., Abreu L.C., Barros E.P.M., Sorpreso I.C.E., Baracat M.C.P., Baracat E.C., Soares Junior J.M. (2019). Repercussions of Melatonin on the Risk of Breast Cancer: A Systematic Review and Meta-Analysis. Rev. Assoc. Médica Bras..

[70] Kalyanaraman B. (2022). NAC, NAC, Knockin’ on Heaven’s Door: Interpreting the Mechanism of Action of N-Acetylcysteine in Tumor and Immune Cells. Redox Biol..

[71] Kindlon E.A., Pidgeon G.P. (2025). N-Acetyl Cysteine as a Promising Therapeutic Approach in Ovarian Cancer: Potential and Perspectives. Acad. Oncol..

[72] Sagristá M.L., GarcÍa A.F., De Madariaga M.A., Mora M. (2002). Antioxidant and Pro-Oxidant Effect of the Thiolic Compounds N -Acetyl- l -Cysteine and Glutathione against Free Radical-Induced Lipid Peroxidation. Free Radic. Res..

[73] Bhosale P.B., Ha S.E., Vetrivel P., Kim H.H., Kim S.M., Kim G.S. (2020). Functions of Polyphenols and Its Anticancer Properties in Biomedical Research: A Narrative Review. Transl. Cancer Res..

[74] Gawlik-Dziki U., Świeca M., Sułkowski M., Dziki D., Baraniak B., Czyż J. (2013). Antioxidant and Anticancer Activities of Chenopodium Quinoa Leaves Extracts—In Vitro Study. Food Chem. Toxicol..

[75] Amaroli A., Panfoli I., Bozzo M., Ferrando S., Candiani S., Ravera S. (2024). The Bright Side of Curcumin: A Narrative Review of Its Therapeutic Potential in Cancer Management. Cancers.

[76] Zoi V., Galani V., Lianos G.D., Voulgaris S., Kyritsis A.P., Alexiou G.A. (2021). The Role of Curcumin in Cancer Treatment. Biomedicines.

[77] Wolnicka-Glubisz A., Wisniewska-Becker A. (2023). Dual Action of Curcumin as an Anti- and Pro-Oxidant from a Biophysical Perspective. Antioxidants.

[78] Serafini M.M., Catanzaro M., Fagiani F., Simoni E., Caporaso R., Dacrema M., Romanoni I., Govoni S., Racchi M., Daglia M. (2020). Modulation of Keap1/Nrf2/ARE Signaling Pathway by Curcuma- and Garlic-Derived Hybrids. Front. Pharmacol..

[79] Baum L., Ng A. (2004). Curcumin Interaction with Copper and Iron Suggests One Possible Mechanism of Action in Alzheimer’s Disease Animal Models. J. Alzheimers Dis..

[80] Chen B., Zhang Y., Wang Y., Rao J., Jiang X., Xu Z. (2014). Curcumin Inhibits Proliferation of Breast Cancer Cells through Nrf2-Mediated down-Regulation of Fen1 Expression. J. Steroid Biochem. Mol. Biol..

[81] Choi H., Chun Y.-S., Kim S.-W., Kim M.-S., Park J.-W. (2006). Curcumin Inhibits Hypoxia-Inducible Factor-1 by Degrading Aryl Hydrocarbon Receptor Nuclear Translocator: A Mechanism of Tumor Growth Inhibition. Mol. Pharmacol..

[82] Ashrafizadeh M., Zarrabi A., Hashemi F., Moghadam E.R., Hashemi F., Entezari M., Hushmandi K., Mohammadinejad R., Najafi M. (2020). Curcumin in Cancer Therapy: A Novel Adjunct for Combination Chemotherapy with Paclitaxel and Alleviation of Its Adverse Effects. Life Sci..

[83] Dhillon N., Aggarwal B.B., Newman R.A., Wolff R.A., Kunnumakkara A.B., Abbruzzese J.L., Ng C.S., Badmaev V., Kurzrock R. (2008). Phase II Trial of Curcumin in Patients with Advanced Pancreatic Cancer. Clin. Cancer Res..

[84] Mansouri K., Rasoulpoor S., Daneshkhah A., Abolfathi S., Salari N., Mohammadi M., Rasoulpoor S., Shabani S. (2020). Clinical Effects of Curcumin in Enhancing Cancer Therapy: A Systematic Review. BMC Cancer.

[85] Santosa D., Suharti C., Riwanto I., Dharmana E., Adhi Pangarsa E., Setiawan B., Suyono S., Lumban Tobing M., Suhartono S., Hadisaputro S. (2022). Curcumin as Adjuvant Therapy to Improve Remission in Myeloma Patients: A Pilot Randomized Clinical Trial. Casp. J. Intern. Med..

[86] Talib W.H., Awajan D., Alqudah A., Alsawwaf R., Althunibat R., Abu AlRoos M., Al Safadi A., Abu Asab S., Hadi R.W., Al Kury L.T. (2024). Targeting Cancer Hallmarks with Epigallocatechin Gallate (EGCG): Mechanistic Basis and Therapeutic Targets. Molecules.

[87] Tang S.-M., Deng X.-T., Zhou J., Li Q.-P., Ge X.-X., Miao L. (2020). Pharmacological Basis and New Insights of Quercetin Action in Respect to Its Anti-Cancer Effects. Biomed. Pharmacother..

[88] Reyes-Farias M., Carrasco-Pozo C. (2019). The Anti-Cancer Effect of Quercetin: Molecular Implications in Cancer Metabolism. Int. J. Mol. Sci..

[89] Lomozová Z., Catapano M.C., Hrubša M., Karlíčková J., Macáková K., Kučera R., Mladěnka P. (2021). Chelation of Iron and Copper by Quercetin B-Ring Methyl Metabolites, Isorhamnetin and Tamarixetin, and Their Effect on Metal-Based Fenton Chemistry. J. Agric. Food Chem..

[90] Cheng M., Yuan C., Ju Y., Liu Y., Shi B., Yang Y., Jin S., He X., Zhang L., Min D. (2024). Quercetin Attenuates Oxidative Stress and Apoptosis in Brain Tissue of APP/PS1 Double Transgenic AD Mice by Regulating Keap1/Nrf2/HO-1 Pathway to Improve Cognitive Impairment. Behav. Neurol..

[91] Zhang Y.-M., Zhang Z.-Y., Wang R.-X. (2020). Protective Mechanisms of Quercetin Against Myocardial Ischemia Reperfusion Injury. Front. Physiol..

[92] Anwar M.J., Altaf A., Imran M., Amir M., Alsagaby S.A., Abdulmonem W.A., Mujtaba A., El-Ghorab A.H., Ghoneim M.M., Hussain M. (2023). Anti-Cancer Perspectives of Resveratrol: A Comprehensive Review. Food Agric. Immunol..

[93] Kursvietiene L., Kopustinskiene D.M., Staneviciene I., Mongirdiene A., Kubová K., Masteikova R., Bernatoniene J. (2023). Anti-Cancer Properties of Resveratrol: A Focus on Its Impact on Mitochondrial Functions. Antioxidants.

[94] Almatroodi S.A., Alsahli M.A., Aljohani A.S.M., Alhumaydhi F.A., Babiker A.Y., Khan A.A., Rahmani A.H. (2022). Potential Therapeutic Targets of Resveratrol, a Plant Polyphenol, and Its Role in the Therapy of Various Types of Cancer. Molecules.

[95] Athar M., Back J., Tang X., Kim K., Kopelovich L., Bickers D., Kim A. (2007). Resveratrol: A Review of Preclinical Studies for Human Cancer Prevention. Toxicol. Appl. Pharmacol..

[96] Meng X., Zhou J., Zhao C.-N., Gan R.-Y., Li H.-B. (2020). Health Benefits and Molecular Mechanisms of Resveratrol: A Narrative Review. Foods.

[97] Belguendouz L., Fremont L., Linard A. (1997). Resveratrol Inhibits Metal Ion-Dependent and Independent Peroxidation of Porcine Low-Density Lipoproteins. Biochem. Pharmacol..

[98] Zini R., Morin C., Bertelli A., Bertelli A.A., Tillement J.P. (1999). Effects of Resveratrol on the Rat Brain Respiratory Chain. Drugs Exp. Clin. Res..

[99] He L., Zhang L., Peng Y., He Z. (2025). Selenium in Cancer Management: Exploring the Therapeutic Potential. Front. Oncol..

[100] Tinggi U. (2008). Selenium: Its Role as Antioxidant in Human Health. Environ. Health Prev. Med..

[101] Ingold I., Berndt C., Schmitt S., Doll S., Poschmann G., Buday K., Roveri A., Peng X., Porto Freitas F., Seibt T. (2018). Selenium Utilization by GPX4 Is Required to Prevent Hydroperoxide-Induced Ferroptosis. Cell.

[102] Xiang N., Zhao R., Zhong W. (2009). Sodium Selenite Induces Apoptosis by Generation of Superoxide via the Mitochondrial-Dependent Pathway in Human Prostate Cancer Cells. Cancer Chemother. Pharmacol..

[103] Lykkesfeldt J., Carr A.C. (2024). Vitamin C. Adv. Nutr..

[104] Zhao H., Fu W., Yang X., Zhang W., Wu S., Ma J., Zhang T., Yao H., Zhang Z. (2026). High-Dose Vitamin C: A Promising Anti-Tumor Agent, Insight from Mechanisms, Clinical Research, and Challenges. Genes. Dis..

[105] Li W.-N., Zhang S.-J., Feng J.-Q., Jin W.-L. (2022). Repurposing Vitamin C for Cancer Treatment: Focus on Targeting the Tumor Microenvironment. Cancers.

[106] Kaźmierczak-Barańska J., Boguszewska K., Adamus-Grabicka A., Karwowski B.T. (2020). Two Faces of Vitamin C—Antioxidative and Pro-Oxidative Agent. Nutrients.

[107] Timoshnikov V.A., Kobzeva T.V., Polyakov N.E., Kontoghiorghes G.J. (2020). Redox Interactions of Vitamin C and Iron: Inhibition of the Pro-Oxidant Activity by Deferiprone. Int. J. Mol. Sci..

[108] Miles S.L., Fischer A.P., Joshi S.J., Niles R.M. (2015). Ascorbic Acid and Ascorbate-2-Phosphate Decrease HIF Activity and Malignant Properties of Human Melanoma Cells. BMC Cancer.

[109] Bodeker K.L., Smith B.J., Berg D.J., Chandrasekharan C., Sharif S., Fei N., Vollstedt S., Brown H., Chandler M., Lorack A. (2024). A Randomized Trial of Pharmacological Ascorbate, Gemcitabine, and Nab-Paclitaxel for Metastatic Pancreatic Cancer. Redox Biol..

[110] Böttger F., Vallés-Martí A., Cahn L., Jimenez C.R. (2021). High-Dose Intravenous Vitamin C, a Promising Multi-Targeting Agent in the Treatment of Cancer. J. Exp. Clin. Cancer Res..

[111] Cameron E., Pauling L. (1976). Supplemental Ascorbate in the Supportive Treatment of Cancer: Prolongation of Survival Times in Terminal Human Cancer. Proc. Natl. Acad. Sci. USA.

[112] Furqan M., Abu-Hejleh T., Stephens L.M., Hartwig S.M., Mott S.L., Pulliam C.F., Petronek M., Henrich J.B., Fath M.A., Houtman J.C. (2022). Pharmacological Ascorbate Improves the Response to Platinum-Based Chemotherapy in Advanced Stage Non-Small Cell Lung Cancer. Redox Biol..

[113] Van Gorkom G.N.Y., Lookermans E.L., Van Elssen C.H.M.J., Bos G.M.J. (2019). The Effect of Vitamin C (Ascorbic Acid) in the Treatment of Patients with Cancer: A Systematic Review. Nutrients.

[114] Wang F., He M.-M., Wang Z.-X., Li S., Jin Y., Ren C., Shi S.-M., Bi B.-T., Chen S.-Z., Lv Z.-D. (2019). Phase I Study of High-Dose Ascorbic Acid with mFOLFOX6 or FOLFIRI in Patients with Metastatic Colorectal Cancer or Gastric Cancer. BMC Cancer.

[115] Creagan E.T., Moertel C.G., O’Fallon J.R., Schutt A.J., O’Connell M.J., Rubin J., Frytak S. (1979). Failure of High-Dose Vitamin C (Ascorbic Acid) Therapy to Benefit Patients with Advanced Cancer: A Controlled Trial. N. Engl. J. Med..

[116] Moertel C.G., Fleming T.R., Creagan E.T., Rubin J., O’Connell M.J., Ames M.M. (1985). High-Dose Vitamin C versus Placebo in the Treatment of Patients with Advanced Cancer Who Have Had No Prior Chemotherapy: A Randomized Double-Blind Comparison. N. Engl. J. Med..

[117] Höbaus J., Thiem U., Hummel D.M., Kallay E. (2013). Role of Calcium, Vitamin D, and the Extrarenal Vitamin D Hydroxylases in Carcinogenesis. Anticancer. Agents Med. Chem..

[118] Muñoz A., Grant W.B. (2022). Vitamin D and Cancer: An Historical Overview of the Epidemiology and Mechanisms. Nutrients.

[119] Martinez P., Grant W.B. (2025). Vitamin D: What Role in Obesity-Related Cancer?. Semin. Cancer Biol..

[120] Li J., Cao Y., Xu J., Li J., Lv C., Gao Q., Zhang C., Jin C., Wang R., Jiao R. (2023). Vitamin D Improves Cognitive Impairment and Alleviates Ferroptosis via the Nrf2 Signaling Pathway in Aging Mice. Int. J. Mol. Sci..

[121] Parvizi Mastali V., Hoseini R., Azizi M. (2023). The Effect of Short-Term Vitamin D on the Antioxidant Capacity Following Exhaustive Aerobic Exercise. Afr. Health Sci..

[122] Ji M.-T., Nie J., Nie X.-F., Hu W.-T., Pei H.-L., Wan J.-M., Wang A.-Q., Zhou G.-M., Zhang Z.-L., Chang L. (2020). 1α,25(OH)2D3 Radiosensitizes Cancer Cells by Activating the NADPH/ROS Pathway. Front. Pharmacol..

[123] Shang Q.-X., Yang Y.-S., Zhang H.-L., Cheng Y.-P., Lu H., Yuan Y., Chen L.-Q., Ji A.-F. (2024). Vitamin D Receptor Induces Oxidative Stress to Promote Esophageal Squamous Cell Carcinoma Proliferation via the P53 Signaling Pathway. Heliyon.

[124] Chen L., Yang R., Qiao W., Yuan X., Wang S., Goltzman D., Miao D. (2018). 1,25-Dihydroxy Vitamin D Prevents Tumorigenesis by Inhibiting Oxidative Stress and Inducing Tumor Cellular Senescence in Mice. Int. J. Cancer.

[125] Kwon C.H., Safaie E.S., Torres J.A., Yang Z., Chen X., Jang Y.D. (2026). Effects of Dietary Vitamin D3 Supplementation on Growth Performance, Blood Vitamin D Status, and Antioxidant Capacity in Weaning Pigs. Anim. Biosci..

[126] Keum N., Lee D.H., Greenwood D.C., Manson J.E., Giovannucci E. (2019). Vitamin D Supplementation and Total Cancer Incidence and Mortality: A Meta-Analysis of Randomized Controlled Trials. Ann. Oncol..

[127] McDonnell S.L., Baggerly C.A., French C.B., Baggerly L.L., Garland C.F., Gorham E.D., Hollis B.W., Trump D.L., Lappe J.M. (2018). Breast Cancer Risk Markedly Lower with Serum 25-Hydroxyvitamin D Concentrations ≥60 vs <20 Ng/ML (150 vs 50 Nmol/L): Pooled Analysis of Two Randomized Trials and a Prospective Cohort. PLoS ONE.

[128] Grant W.B., Boucher B.J., Al Anouti F., Pilz S. (2022). Comparing the Evidence from Observational Studies and Randomized Controlled Trials for Nonskeletal Health Effects of Vitamin D. Nutrients.

[129] Manson J.E., Cook N.R., Lee I.-M., Christen W., Bassuk S.S., Mora S., Gibson H., Gordon D., Copeland T., D’Agostino D. (2019). Vitamin D Supplements and Prevention of Cancer and Cardiovascular Disease. N. Engl. J. Med..

[130] Niedermaier T., Gredner T., Kuznia S., Schöttker B., Mons U., Brenner H. (2021). Vitamin D Supplementation to the Older Adult Population in Germany Has the Cost-saving Potential of Preventing Almost 30,000 Cancer Deaths per Year. Mol. Oncol..

[131] Es-Sai B., Wahnou H., Benayad S., Rabbaa S., Laaziouez Y., El Kebbaj R., Limami Y., Duval R.E. (2025). Gamma-Tocopherol: A Comprehensive Review of Its Antioxidant, Anti-Inflammatory, and Anticancer Properties. Molecules.

[132] Venugopal S.K., Devaraj S., Yang T., Jialal I. (2002). α-Tocopherol Decreases Superoxide Anion Release in Human Monocytes Under Hyperglycemic Conditions Via Inhibition of Protein Kinase C-α. Diabetes.

[133] Yeganehjoo H., DeBose-Boyd R., McFarlin B.K., Mo H. (2017). Synergistic Impact of *d* -δ-Tocotrienol and Geranylgeraniol on the Growth and HMG CoA Reductase of Human DU145 Prostate Carcinoma Cells. Nutr. Cancer.

[134] Ye C., Zhao W., Li M., Zhuang J., Yan X., Lu Q., Chang C., Huang X., Zhou J., Xie B. (2015). δ-Tocotrienol Induces Human Bladder Cancer Cell Growth Arrest, Apoptosis and Chemosensitization through Inhibition of STAT3 Pathway. PLoS ONE.

[135] Bjelakovic G., Nikolova D., Gluud L.L., Simonetti R.G., Gluud C. (2007). Mortality in Randomized Trials of Antioxidant Supplements for Primary and Secondary Prevention: Systematic Review and Meta-Analysis. JAMA.

[136] Yang C.S., Luo P., Zeng Z., Wang H., Malafa M., Suh N. (2020). Vitamin E and Cancer Prevention: Studies with Different Forms of Tocopherols and Tocotrienols. Mol. Carcinog..

[137] Wright M.E., Virtamo J., Hartman A.M., Pietinen P., Edwards B.K., Taylor P.R., Huttunen J.K., Albanes D. (2007). Effects of α-tocopherol and β-carotene Supplementation on Upper Aerodigestive Tract Cancers in a Large, Randomized Controlled Trial. Cancer.

[138] Bjelakovic G., Nikolova D., Simonetti R.G., Gluud C. (2004). Antioxidant Supplements for Prevention of Gastrointestinal Cancers: A Systematic Review and Meta-Analysis. Lancet.

[139] Bardia A., Tleyjeh I.M., Cerhan J.R., Sood A.K., Limburg P.J., Erwin P.J., Montori V.M. (2008). Efficacy of Antioxidant Supplementation in Reducing Primary Cancer Incidence and Mortality: Systematic Review and Meta-Analysis. Mayo Clin. Proc..

[140] Myung S.-K., Kim Y., Ju W., Choi H.J., Bae W.K. (2010). Effects of Antioxidant Supplements on Cancer Prevention: Meta-Analysis of Randomized Controlled Trials. Ann. Oncol..

[141] Omenn G.S., Goodman G.E., Thornquist M.D., Balmes J., Cullen M.R., Glass A., Keogh J.P., Meyskens F.L., Valanis B., Williams J.H. (1996). Risk Factors for Lung Cancer and for Intervention Effects in CARET, the Beta-Carotene and Retinol Efficacy Trial. JNCI J. Natl. Cancer Inst..

[142] Zhang Y., Yang J., Na X., Zhao A. (2023). Association between β-Carotene Supplementation and Risk of Cancer: A Meta-Analysis of Randomized Controlled Trials. Nutr. Rev..

[143] Vinceti M., Filippini T., Del Giovane C., Dennert G., Zwahlen M., Brinkman M., Zeegers M.P., Horneber M., D’Amico R., Crespi C.M. (2018). Selenium for Preventing Cancer. Cochrane Database Syst. Rev..

[144] Qiao Y.-L., Dawsey S.M., Kamangar F., Fan J.-H., Abnet C.C., Sun X.-D., Johnson L.L., Gail M.H., Dong Z.-W., Yu B. (2009). Total and Cancer Mortality After Supplementation with Vitamins and Minerals: Follow-up of the Linxian General Population Nutrition Intervention Trial. JNCI J. Natl. Cancer Inst..

[145] Yasueda A., Urushima H., Ito T. (2016). Efficacy and Interaction of Antioxidant Supplements as Adjuvant Therapy in Cancer Treatment: A Systematic Review. Integr. Cancer Ther..

[146] Li Y., Lin Q., Lu X., Li W. (2021). Post-Diagnosis Use of Antioxidant Vitamin Supplements and Breast Cancer Prognosis: A Systematic Review and Meta-Analysis. Clin. Breast Cancer.

[147] Psaltopoulou T., Ntanasis-Stathopoulos I., Tsilimigras D.I., Tzanninis I.-G., Gavriatopoulou M., Sergentanis T.N. (2018). Micronutrient Intake and Risk of Hematological Malignancies in Adults: A Systematic Review and Meta-Analysis of Cohort Studies. Nutr. Cancer.

[148] Hercberg S., Kesse-Guyot E., Druesne-Pecollo N., Touvier M., Favier A., Latino-Martel P., Briançon S., Galan P. (2010). Incidence of Cancers, Ischemic Cardiovascular Diseases and Mortality during 5-year Follow-up after Stopping Antioxidant Vitamins and Minerals Supplements: A Postintervention Follow-up in the SU.VI.MAX Study. Int. J. Cancer.

[149] Gaziano J.M., Glynn R.J., Christen W.G., Kurth T., Belanger C., MacFadyen J., Bubes V., Manson J.E., Sesso H.D., Buring J.E. (2009). Vitamins E and C in the Prevention of Prostate and Total Cancer in Men: The Physicians’ Health Study II Randomized Controlled Trial. JAMA.

[150] Arshadi M., Ghazal N., Ghavidel F., Beygi Z., Nasiri Z., Zarepour P., Abdollahi S., Azizi H., Khodamoradi F. (2025). The Association between Vitamin C and Breast Cancer, Prostate Cancer and Colorectal Cancer: A Systematic Review and Meta-Analysis. Clin. Nutr. ESPEN.

[151] Lee B., Oh S.-W., Myung S.-K. (2015). Efficacy of Vitamin C Supplements in Prevention of Cancer: A Meta-Analysis of Randomized Controlled Trials. Korean J. Fam. Med..

[152] Chen Z., Huang Y., Cao D., Qiu S., Chen B., Li J., Bao Y., Wei Q., Han P., Liu L. (2022). Vitamin C Intake and Cancers: An Umbrella Review. Front. Nutr..

[153] Zhang D., Xu P., Li Y., Wei B., Yang S., Zheng Y., Lyu L., Deng Y., Zhai Z., Li N. (2020). Association of Vitamin C Intake with Breast Cancer Risk and Mortality: A Meta-Analysis of Observational Studies. Aging.

[154] Aune D., Keum N., Giovannucci E., Fadnes L.T., Boffetta P., Greenwood D.C., Tonstad S., Vatten L.J., Riboli E., Norat T. (2018). Dietary Intake and Blood Concentrations of Antioxidants and the Risk of Cardiovascular Disease, Total Cancer, and All-Cause Mortality: A Systematic Review and Dose-Response Meta-Analysis of Prospective Studies. Am. J. Clin. Nutr..

[155] Vaughan-Shaw P.G., Buijs L.F., Blackmur J.P., Theodoratou E., Zgaga L., Din F.V.N., Farrington S.M., Dunlop M.G. (2020). The Effect of Vitamin D Supplementation on Survival in Patients with Colorectal Cancer: Systematic Review and Meta-Analysis of Randomised Controlled Trials. Br. J. Cancer.

[156] Lee I.-M., Cook N.R., Gaziano J.M., Gordon D., Ridker P.M., Manson J.E., Hennekens C.H., Buring J.E. (2005). Vitamin E in the Primary Prevention of Cardiovascular Disease and Cancer: The Women’s Health Study: A Randomized Controlled Trial. JAMA.

[157] Chan S., Xiong P., Zhao M., Zhang S., Zheng R., Ye J., Chan K.I., Li C., Zhong Z. (2024). Anti-inflammatory Effects of Natural Products from Vitamin C-rich Fruits. Food Front..

[158] Grant W., Wimalawansa S., Pludowski P., Cheng R. (2025). Vitamin D: Evidence-Based Health Benefits and Recommendations for Population Guidelines. Nutrients.

[159] Davies M., Mawer E.B., Krawitt E.L. (1980). Comparative Absorption of Vitamin D3 and 25-Hydroxyvitamin D3 in Intestinal Disease. Gut.

[160] Thompson G.R., Lewis B., Booth C.C. (1966). Absorption of Vitamin D3-3H in Control Subjects and Patients with Intestinal Malabsorption. J. Clin. Investig..

[161] Ng K., Nimeiri H.S., McCleary N.J., Abrams T.A., Yurgelun M.B., Cleary J.M., Rubinson D.A., Schrag D., Miksad R., Bullock A.J. (2019). Effect of High-Dose vs Standard-Dose Vitamin D_3_ Supplementation on Progression-Free Survival Among Patients with Advanced or Metastatic Colorectal Cancer: The SUNSHINE Randomized Clinical Trial. JAMA.

[162] Hermann R., Niebch G., Borbe H.O., Fieger-Büschges H., Ruus P., Nowak H., Riethmüller-Winzen H., Peukert M., Blume H. (1996). Enantioselective Pharmacokinetics and Bioavailability of Different Racemic α-Lipoic Acid Formulations in Healthy Volunteers. Eur. J. Pharm. Sci..

[163] Fairweather-Tait S.J., Collings R., Hurst R. (2010). Selenium Bioavailability: Current Knowledge and Future Research Requirements. Am. J. Clin. Nutr..

[164] Haskell M.J. (2012). The Challenge to Reach Nutritional Adequacy for Vitamin A: β-Carotene Bioavailability and Conversion—Evidence in Humans. Am. J. Clin. Nutr..

[165] Levine M., Conry-Cantilena C., Wang Y., Welch R.W., Washko P.W., Dhariwal K.R., Park J.B., Lazarev A., Graumlich J.F., King J. (1996). Vitamin C Pharmacokinetics in Healthy Volunteers: Evidence for a Recommended Dietary Allowance. Proc. Natl. Acad. Sci. USA.

[166] Carr A.C. (2025). Do Liposomal Vitamin C Formulations Have Improved Bioavailability? A Scoping Review Identifying Future Research Directions. Basic Clin. Pharmacol. Toxicol..

[167] Hoffer L.J., Robitaille L., Zakarian R., Melnychuk D., Kavan P., Agulnik J., Cohen V., Small D., Miller W.H. (2015). High-Dose Intravenous Vitamin C Combined with Cytotoxic Chemotherapy in Patients with Advanced Cancer: A Phase I-II Clinical Trial. PLoS ONE.

[168] Stephenson C.M., Levin R.D., Spector T., Lis C.G. (2013). Phase I Clinical Trial to Evaluate the Safety, Tolerability, and Pharmacokinetics of High-Dose Intravenous Ascorbic Acid in Patients with Advanced Cancer. Cancer Chemother. Pharmacol..

[169] DeMuro R.L., Nafziger A.N., Blask D.E., Menhinick A.M., Bertino J.S. (2000). The Absolute Bioavailability of Oral Melatonin. J. Clin. Pharmacol..

[170] Harpsøe N.G., Andersen L.P.H., Gögenur I., Rosenberg J. (2015). Clinical Pharmacokinetics of Melatonin: A Systematic Review. Eur. J. Clin. Pharmacol..

[171] Walle T., Hsieh F., DeLegge M.H., Oatis J.E., Walle U.K. (2004). HIGH ABSORPTION BUT VERY LOW BIOAVAILABILITY OF ORAL RESVERATROL IN HUMANS. Drug Metab. Dispos..

[172] Gambini J., Inglés M., Olaso G., Lopez-Grueso R., Bonet-Costa V., Gimeno-Mallench L., Mas-Bargues C., Abdelaziz K.M., Gomez-Cabrera M.C., Vina J. (2015). Properties of Resveratrol: In Vitro and In Vivo Studies about Metabolism, Bioavailability, and Biological Effects in Animal Models and Humans. Oxid. Med. Cell. Longev..

[173] Bruno R.S., Leonard S.W., Park S., Zhao Y., Traber M.G. (2006). Human Vitamin E Requirements Assessed with the Use of Apples Fortified with Deuterium-Labeled α-Tocopheryl Acetate. Am. J. Clin. Nutr..

[174] Hua W., Ding L., Chen Y., Gong B., He J., Xu G. (2007). Determination of Berberine in Human Plasma by Liquid Chromatography–Electrospray Ionization–Mass Spectrometry. J. Pharm. Biomed. Anal..

[175] Wang A., Yang W., Yang X., Mei X., Hu T., Liang R., Meng D., Yan D. (2020). MgAl Monolayer Hydrotalcite Increases the Hypoglycemic Effect of Berberine by Enhancing Its Oral Bioavailability. Biomed. Pharmacother..

[176] Judy W.V. (2022). The Single-Dose Absorption and Steady-State Bioavailability of Different Coenzyme Q10 Formulations. Integr. Med. Encinitas Calif..

[177] Bhagavan H.N., Chopra R.K. (2006). Coenzyme Q10: Absorption, Tissue Uptake, Metabolism and Pharmacokinetics. Free Radic. Res..

[178] Kroon M.A.G.M., Van Laarhoven H.W.M., Swart E.L., Van Tellingen O., Kemper E.M. (2025). A Pharmacokinetic Study and Critical Reappraisal of Curcumin Formulations Enhancing Bioavailability. iScience.

[179] Henning S.M., Niu Y., Lee N.H., Thames G.D., Minutti R.R., Wang H., Go V.L.W., Heber D. (2004). Bioavailability and Antioxidant Activity of Tea Flavanols after Consumption of Green Tea, Black Tea, or a Green Tea Extract Supplement. Am. J. Clin. Nutr..

[180] Olsson B., Johansson M., Gabrielsson J., Bolme P. (1988). Pharmacokinetics and Bioavailability of Reduced and Oxidized N-Acetylcysteine. Eur. J. Clin. Pharmacol..

[181] Borgström L., Kågedal B., Paulsen O. (1986). Pharmacokinetics of N-Acetylcysteine in Man. Eur. J. Clin. Pharmacol..

[182] Kandemir K., Tomas M., McClements D.J., Capanoglu E. (2022). Recent Advances on the Improvement of Quercetin Bioavailability. Trends Food Sci. Technol..

[183] Warias P., Plewa P., Poniewierska-Baran A. (2024). Resveratrol, Piceatannol, Curcumin, and Quercetin as Therapeutic Targets in Gastric Cancer—Mechanisms and Clinical Implications for Natural Products. Molecules.

[184] Seyfried T.N., Lee D.C., Duraj T., Ta N.L., Mukherjee P., Kiebish M., Arismendi-Morillo G., Chinopoulos C. (2025). The Warburg Hypothesis and the Emergence of the Mitochondrial Metabolic Theory of Cancer. J. Bioenerg. Biomembr..

[185] Martinez P., Baghli I., Gourjon G., Seyfried T.N. (2024). Mitochondrial–Stem Cell Connection: Providing Additional Explanations for Understanding Cancer. Metabolites.

[186] Lee D.C., Ta L., Mukherjee P., Duraj T., Domin M., Greenwood B., Karmacharya S., Narain N.R., Kiebish M., Chinopoulos C. (2024). Amino Acid and Glucose Fermentation Maintain ATP Content in Mouse and Human Malignant Glioma Cells. ASN Neuro.

[187] Fernandez-de-Cossio-Diaz J., Vazquez A. (2017). Limits of Aerobic Metabolism in Cancer Cells. Sci. Rep..

[188] Taylor C.T. (2008). Mitochondria and Cellular Oxygen Sensing in the HIF Pathway. Biochem. J..

[189] Chinopoulos C. (2026). Mitochondrial Respiration Supports Cancer Growth Independent of OXPHOS. Biochim. Biophys. Acta BBA—Rev. Cancer.

[190] Kozlov A.V., Gille L., Staniek K., Nohl H. (1999). Dihydrolipoic Acid Maintains Ubiquinone in the Antioxidant Active Form by Two-Electron Reduction of Ubiquinone and One-Electron Reduction of Ubisemiquinone. Arch. Biochem. Biophys..

[191] Mukha D., Fokra M., Feldman A., Sarvin B., Sarvin N., Nevo-Dinur K., Besser E., Hallo E., Aizenshtein E., Schug Z.T. (2022). Glycine Decarboxylase Maintains Mitochondrial Protein Lipoylation to Support Tumor Growth. Cell Metab..

[192] Solmonson A., DeBerardinis R.J. (2018). Lipoic Acid Metabolism and Mitochondrial Redox Regulation. J. Biol. Chem..

[193] Ming M., Sinnett-Smith J., Wang J., Soares H.P., Young S.H., Eibl G., Rozengurt E. (2014). Dose-Dependent AMPK-Dependent and Independent Mechanisms of Berberine and Metformin Inhibition of mTORC1, ERK, DNA Synthesis and Proliferation in Pancreatic Cancer Cells. PLoS ONE.

[194] Huang Z., Shen Y., Liu W., Yang Y., Guo L., Yan Q., Wei C., Guo Q., Fan X., Ma W. (2023). Berberine Targets the Electron Transport Chain Complex I and Reveals the Landscape of OXPHOS Dependency in Acute Myeloid Leukemia with IDH1 Mutation. Chin. J. Nat. Med..

[195] Yan X.-J., Yu X., Wang X.-P., Jiang J.-F., Yuan Z.-Y., Lu X., Lei F., Xing D.-M. (2017). Mitochondria Play an Important Role in the Cell Proliferation Suppressing Activity of Berberine. Sci. Rep..

[196] López-Martín J.M., Salviati L., Trevisson E., Montini G., DiMauro S., Quinzii C., Hirano M., Rodriguez-Hernandez A., Cordero M.D., Sánchez-Alcázar J.A. (2007). Missense Mutation of the COQ2 Gene Causes Defects of Bioenergetics and de Novo Pyrimidine Synthesis. Hum. Mol. Genet..

[197] González-García P., Hidalgo-Gutiérrez A., Mascaraque C., Barriocanal-Casado E., Bakkali M., Ziosi M., Abdihankyzy U.B., Sánchez-Hernández S., Escames G., Prokisch H. (2020). Coenzyme Q10 Modulates Sulfide Metabolism and Links the Mitochondrial Respiratory Chain to Pathways Associated to One Carbon Metabolism. Hum. Mol. Genet..

[198] Grayson C., Mailloux R.J. (2023). Coenzyme Q10 and Nicotinamide Nucleotide Transhydrogenase: Sentinels for Mitochondrial Hydrogen Peroxide Signaling. Free Radic. Biol. Med..

[199] Chashmniam S., Mirhafez S.R., Dehabeh M., Hariri M., Azimi Nezhad M., Nobakht M., Gh B.F. (2019). A Pilot Study of the Effect of Phospholipid Curcumin on Serum Metabolomic Profile in Patients with Non-Alcoholic Fatty Liver Disease: A Randomized, Double-Blind, Placebo-Controlled Trial. Eur. J. Clin. Nutr..

[200] Fan Y., Xue H., Li Z., Huo M., Gao H., Guan X. (2024). Exploiting the Achilles’ Heel of Cancer: Disrupting Glutamine Metabolism for Effective Cancer Treatment. Front. Pharmacol..

[201] Peeters T.H., Lenting K., Breukels V., Van Lith S.A.M., Van Den Heuvel C.N.A.M., Molenaar R., Van Rooij A., Wevers R., Span P.N., Heerschap A. (2019). Isocitrate Dehydrogenase 1-Mutated Cancers Are Sensitive to the Green Tea Polyphenol Epigallocatechin-3-Gallate. Cancer Metab..

[202] Yang G., Shan Y., Zhang Z., Wu H., Lei Y., Sun Y., Ji P., Guoshi L. (2025). Melatonin Rescues Heat Stress-Induced Suppression of TCA Cycle and Mitochondrial Damage in Goat Sertoli Cells. Int. J. Mol. Sci..

[203] Ezeriņa D., Takano Y., Hanaoka K., Urano Y., Dick T.P. (2018). N-Acetyl Cysteine Functions as a Fast-Acting Antioxidant by Triggering Intracellular H2S and Sulfane Sulfur Production. Cell Chem. Biol..

[204] Amador-Martínez I., Aparicio-Trejo O.E., Aranda-Rivera A.K., Bernabe-Yepes B., Medina-Campos O.N., Tapia E., Cortés-González C.C., Silva-Palacios A., Roldán F.J., León-Contreras J.C. (2025). Effect of N-Acetylcysteine in Mitochondrial Function, Redox Signaling, and Sirtuin 3 Levels in the Heart During Cardiorenal Syndrome Type 4 Development. Antioxidants.

[205] Desquiret-Dumas V., Gueguen N., Leman G., Baron S., Nivet-Antoine V., Chupin S., Chevrollier A., Vessières E., Ayer A., Ferré M. (2013). Resveratrol Induces a Mitochondrial Complex I-Dependent Increase in NADH Oxidation Responsible for Sirtuin Activation in Liver Cells. J. Biol. Chem..

[206] Lee N., Park S.J., Lange M., Tseyang T., Doshi M.B., Kim T.Y., Song Y., Kim D.I., Greer P.L., Olzmann J.A. (2024). Selenium Reduction of Ubiquinone via SQOR Suppresses Ferroptosis. Nat. Metab..

[207] Uetaki M., Tabata S., Nakasuka F., Soga T., Tomita M. (2015). Metabolomic Alterations in Human Cancer Cells by Vitamin C-Induced Oxidative Stress. Sci. Rep..

[208] Long Y., Qiu J., Zhang B., He P., Shi X., He Q., Chen Z., Shen W., Li Z., Zhang X. (2021). Pharmacological Vitamin C Treatment Impedes the Growth of Endogenous Glutamine-Dependent Cancers by Targeting Glutamine Synthetase. Front. Pharmacol..

[209] Consiglio M., Destefanis M., Morena D., Foglizzo V., Forneris M., Pescarmona G., Silvagno F. (2014). The Vitamin D Receptor Inhibits the Respiratory Chain, Contributing to the Metabolic Switch That Is Essential for Cancer Cell Proliferation. PLoS ONE.

[210] Kanwal B., Pounraj S., Hanif R., Kovacevic Z. (2026). Metabolic Crosstalk in Triple-Negative Breast Cancer Lung Metastasis: Differential Effects of Vitamin D and E in a Co-Culture System. Cancers.

[211] Calhoon D., Sang L., Ji F., Bezwada D., Hsu S.-C., Cai F., Kim N., Basu A., Wu R., Pimentel A. (2025). Glycosaminoglycan-Driven Lipoprotein Uptake Protects Tumours from Ferroptosis. Nature.

[212] Yeh H., DelGaudio N.L., Uygur B., Millet A., Khan A., Unlu G., Xiao M., Timson R.C., Li C., Ozcan K. (2025). Mitochondrial Glutathione Import Enables Breast Cancer Metastasis via Integrated Stress Response Signaling. Cancer Discov..

[213] Traverso N., Ricciarelli R., Nitti M., Marengo B., Furfaro A.L., Pronzato M.A., Marinari U.M., Domenicotti C. (2013). Role of Glutathione in Cancer Progression and Chemoresistance. Oxid. Med. Cell. Longev..

[214] Zhang Z., Gao J., Jia L., Kong S., Zhai M., Wang S., Li W., Wang S., Su Y., Li W. (2024). Excessive Glutathione Intake Contributes to Chemotherapy Resistance in Breast Cancer: A Propensity Score Matching Analysis. World J. Surg. Oncol..

[215] Sayin V.I., Ibrahim M.X., Larsson E., Nilsson J.A., Lindahl P., Bergo M.O. (2014). Antioxidants Accelerate Lung Cancer Progression in Mice. Sci. Transl. Med..

[216] Wang T., Dong Y., Huang Z., Zhang G., Zhao Y., Yao H., Hu J., Tüksammel E., Cai H., Liang N. (2023). Antioxidants Stimulate BACH1-Dependent Tumor Angiogenesis. J. Clin. Investig..

[217] Le Gal K., Ibrahim M.X., Wiel C., Sayin V.I., Akula M.K., Karlsson C., Dalin M.G., Akyürek L.M., Lindahl P., Nilsson J. (2015). Antioxidants Can Increase Melanoma Metastasis in Mice. Sci. Transl. Med..

[218] Wiel C., Le Gal K., Ibrahim M.X., Jahangir C.A., Kashif M., Yao H., Ziegler D.V., Xu X., Ghosh T., Mondal T. (2019). BACH1 Stabilization by Antioxidants Stimulates Lung Cancer Metastasis. Cell.

[219] Sen U., Shenoy P.S., Bose B. (2017). Opposing Effects of Low versus High Concentrations of Water Soluble Vitamins/Dietary Ingredients Vitamin C and Niacin on Colon Cancer Stem Cells (CSCs). Cell Biol. Int..

[220] Yang G., Yan Y., Ma Y., Yang Y. (2017). Vitamin C at High Concentrations Induces Cytotoxicity in Malignant Melanoma but Promotes Tumor Growth at Low Concentrations. Mol. Carcinog..

[221] Berridge M.J. (2015). Vitamin D Cell Signalling in Health and Disease. Biochem. Biophys. Res. Commun..

[222] Moratilla-Rivera I., Sánchez M., Valdés-González J.A., Gómez-Serranillos M.P. (2023). Natural Products as Modulators of Nrf2 Signaling Pathway in Neuroprotection. Int. J. Mol. Sci..

[223] Xu I.M.-J., Lai R.K.-H., Lin S.-H., Tse A.P.-W., Chiu D.K.-C., Koh H.-Y., Law C.-T., Wong C.-M., Cai Z., Wong C.C.-L. (2016). Transketolase Counteracts Oxidative Stress to Drive Cancer Development. Proc. Natl. Acad. Sci. USA.

[224] Zhou S., Wu H., Ning W., Wu X., Xu X., Ma Y., Li X., Hu J., Wang C., Wang J. (2021). Ivermectin Has New Application in Inhibiting Colorectal Cancer Cell Growth. Front. Pharmacol..

[225] Feng H., He L., Umar T., Wang X., Li W., Zhang B., Zhu X., Deng G., Qiu C. (2025). Synergistic Antitumor Effects of Ivermectin and Metformin in Canine Breast Cancer via PI3K/AKT/mTOR Pathway Inhibition. Curr. Issues Mol. Biol..

[226] Liang Z., Chen Q., Pan L., She X., Chen T. (2024). Mebendazole Induces Apoptosis and Inhibits Migration via the Reactive Oxygen Species-Mediated STAT3 Signaling Downregulation in Non-Small Cell Lung Cancer. J. Thorac. Dis..

[227] Chen P., Stone J., Sullivan G., Drisko J.A., Chen Q. (2011). Anti-Cancer Effect of Pharmacologic Ascorbate and Its Interaction with Supplementary Parenteral Glutathione in Preclinical Cancer Models. Free Radic. Biol. Med..

[228] Zhao H., Duan Q., Zhang Z., Li H., Wu H., Shen Q., Wang C., Yin T. (2017). Up-regulation of Glycolysis Promotes the Stemness and EMT Phenotypes in Gemcitabine-resistant Pancreatic Cancer Cells. J. Cell. Mol. Med..

[229] Singh K., Bhori M., Kasu Y.A., Bhat G., Marar T. (2018). Antioxidants as Precision Weapons in War against Cancer Chemotherapy Induced Toxicity—Exploring the Armoury of Obscurity. Saudi Pharm. J..

[230] Block K.I., Koch A.C., Mead M.N., Tothy P.K., Newman R.A., Gyllenhaal C. (2007). Impact of Antioxidant Supplementation on Chemotherapeutic Efficacy: A Systematic Review of the Evidence from Randomized Controlled Trials. Cancer Treat. Rev..

[231] Ladas E.J., Jacobson J.S., Kennedy D.D., Teel K., Fleischauer A., Kelly K.M. (2004). Antioxidants and Cancer Therapy: A Systematic Review. J. Clin. Oncol..

[232] Ambrosone C.B., Zirpoli G.R., Hutson A.D., McCann W.E., McCann S.E., Barlow W.E., Kelly K.M., Cannioto R., Sucheston-Campbell L.E., Hershman D.L. (2020). Dietary Supplement Use During Chemotherapy and Survival Outcomes of Patients with Breast Cancer Enrolled in a Cooperative Group Clinical Trial (SWOG S0221). J. Clin. Oncol..

[233] Wang M., Xiao Y., Miao J., Zhang X., Liu M., Zhu L., Liu H., Shen X., Wang J., Xie B. (2025). Oxidative Stress and Inflammation: Drivers of Tumorigenesis and Therapeutic Opportunities. Antioxidants.

[234] Chen Q., Espey M.G., Sun A.Y., Pooput C., Kirk K.L., Krishna M.C., Khosh D.B., Drisko J., Levine M. (2008). Pharmacologic Doses of Ascorbate Act as a Prooxidant and Decrease Growth of Aggressive Tumor Xenografts in Mice. Proc. Natl. Acad. Sci. USA.

[235] Chen Q., Espey M.G., Krishna M.C., Mitchell J.B., Corpe C.P., Buettner G.R., Shacter E., Levine M. (2005). Pharmacologic Ascorbic Acid Concentrations Selectively Kill Cancer Cells: Action as a pro-Drug to Deliver Hydrogen Peroxide to Tissues. Proc. Natl. Acad. Sci. USA.

[236] Yun J., Mullarky E., Lu C., Bosch K.N., Kavalier A., Rivera K., Roper J., Chio I.I.C., Giannopoulou E.G., Rago C. (2015). Vitamin C Selectively Kills *KRAS* and *BRAF* Mutant Colorectal Cancer Cells by Targeting GAPDH. Science.

[237] Pinto-Garcia L., Efferth T., Torres A., Hoheisel J., Youns M. (2010). Berberine Inhibits Cell Growth and Mediates Caspase-Independent Cell Death in Human Pancreatic Cancer Cells. Planta Med..

[238] Agnarelli A., Natali M., Garcia-Gil M., Pesi R., Tozzi M.G., Ippolito C., Bernardini N., Vignali R., Batistoni R., Bianucci A.M. (2018). Cell-Specific Pattern of Berberine Pleiotropic Effects on Different Human Cell Lines. Sci. Rep..

[239] Kim H.K., Lee R.-A., Hong K.S., Noh G.T., Oh B.Y. (2025). Cancer-Specific Cytotoxicity of Curcumin through Regulation of Integrin Β1 Expression in Colon Cancer. Sci. Rep..

[240] Wenzel U., Nickel A., Daniel H. (2005). α-Lipoic Acid Induces Apoptosis in Human Colon Cancer Cells by Increasing Mitochondrial Respiration with a Concomitant O^2−^·-Generation. Apoptosis.

[241] Zulkapli R., Abdul Razak F., Zain R.B. (2017). Vitamin E (α-Tocopherol) Exhibits Antitumour Activity on Oral Squamous Carcinoma Cells ORL-48. Integr. Cancer Ther..

[242] Sowmya P.R.-R., Arathi B.P., Vijay K., Baskaran V., Lakshminarayana R. (2017). Astaxanthin from Shrimp Efficiently Modulates Oxidative Stress and Allied Cell Death Progression in MCF-7 Cells Treated Synergistically with β-Carotene and Lutein from Greens. Food Chem. Toxicol..

[243] Mao L., Chen Q., Gong K., Xu X., Xie Y., Zhang W., Cao H., Hu T., Hong X., Zhan Y. (2018). Berberine Decelerates Glucose Metabolism via Suppression of mTOR-dependent HIF-1α Protein Synthesis in Colon Cancer Cells. Oncol. Rep..

[244] Yan X., Yuan C., Wang Z., Xu Z., Wu Z., Wang M., Xu M., Wang Z., Sun Y. (2024). Berberine Modulates Ovarian Cancer Autophagy and Glycolysis through the LINC01123/P65/MAPK10 Signaling Axis. Phytomedicine.

[245] Zhang P., Wang Q., Lin Z., Yang P., Dou K., Zhang R. (2019). Berberine Inhibits Growth of Liver Cancer Cells by Suppressing Glutamine Uptake. OncoTargets Ther..

[246] Yi Y., Wang G., Zhang W., Yu S., Fei J., An T., Yi J., Li F., Huang T., Yang J. (2025). Mitochondrial-Cytochrome c Oxidase II Promotes Glutaminolysis to Sustain Tumor Cell Survival upon Glucose Deprivation. Nat. Commun..

[247] Liparulo I., Bergamini C., Bortolus M., Calonghi N., Gasparre G., Kurelac I., Masin L., Rizzardi N., Rugolo M., Wang W. (2021). Coenzyme Q Biosynthesis Inhibition Induces HIF-1α Stabilization and Metabolic Switch toward Glycolysis. FEBS J..

[248] Wang K., Fan H., Chen Q., Ma G., Zhu M., Zhang X., Zhang Y., Yu J. (2015). Curcumin Inhibits Aerobic Glycolysis and Induces Mitochondrial-Mediated Apoptosis through Hexokinase II in Human Colorectal Cancer Cells in Vitro. Anticancer. Drugs.

[249] Choi D., Lee J.G., Heo S.-H., Cho M.-K., Nam H.-S., Lee S.-H., Lee Y.-J. (2024). Curcumin and Its Potential to Target the Glycolytic Behavior of Lactate-Acclimated Prostate Carcinoma Cells with Docetaxel. Nutrients.

[250] Siddiqui F.A., Prakasam G., Chattopadhyay S., Rehman A.U., Padder R.A., Ansari M.A., Irshad R., Mangalhara K., Bamezai R.N.K., Husain M. (2018). Curcumin Decreases Warburg Effect in Cancer Cells by Down-Regulating Pyruvate Kinase M2 via mTOR-HIF1α Inhibition. Sci. Rep..

[251] Fan W., Wang F., Jin Z., Zhu L., Zhang J. (2022). Curcumin Synergizes with Cisplatin to Inhibit Colon Cancer through Targeting the MicroRNA-137-Glutaminase Axis. Curr. Med. Sci..

[252] Thongpon P., Intuyod K., Chomwong S., Pongking T., Klungsaeng S., Muisuk K., Charoenram N., Sitthirach C., Thanan R., Pinlaor P. (2024). Curcumin Synergistically Enhances the Efficacy of Gemcitabine against Gemcitabine-Resistant Cholangiocarcinoma via the Targeting LAT2/Glutamine Pathway. Sci. Rep..

[253] Shen D., Deng Z., Liu W., Zhou F., Fang Y., Shan D., Wang G., Qian K., Yu M., Zhang Y. (2023). Melatonin Inhibits Bladder Tumorigenesis by Suppressing PPARγ/ENO1-Mediated Glycolysis. Cell Death Dis..

[254] Guerra-Librero A., Fernandez-Gil B.I., Florido J., Martinez-Ruiz L., Rodríguez-Santana C., Shen Y.-Q., García-Verdugo J.M., López-Rodríguez A., Rusanova I., Quiñones-Hinojosa A. (2021). Melatonin Targets Metabolism in Head and Neck Cancer Cells by Regulating Mitochondrial Structure and Function. Antioxidants.

[255] Lai Y.-W., Chu C.-Y., Liu Z.-W., Lu H.-Y., Lee C.-C., Lin M.-H., Lin C.-W. (2026). Melatonin Suppresses Glycolysis and Coordinately Disrupts DNA Repair via Targeting the YAP1-NAMPT Signaling in Breast Cancer. Chem. Biol. Interact..

[256] Silveira H.S., Cesário R.C., Vígaro R.A., Gaiotte L.B., Cucielo M.S., Guimarães F., Seiva F.R.F., Zuccari D.A.P.C., Reiter R.J., Chuffa L.G.D.A. (2024). Melatonin Changes Energy Metabolism and Reduces Oncogenic Signaling in Ovarian Cancer Cells. Mol. Cell. Endocrinol..

[257] Zhao Q., Zhang H., Wu H.-M., Yang Q.-Y., Zhao H., Kang L., Lv X.-Y. (2025). Melatonin-Induced Ferroptosis in Pancreatic Cancer Cells by Stimulating Endoplasmic Reticulum Stress and Inhibiting Alanine-Serine-Cysteine Transporter 2-Driven Glutamine Metabolism. World J. Gastroenterol..

[258] Hevia D., González-Menéndez P., Quiros-González I., Miar A., Rodríguez-García A., Tan D., Reiter R.J., Mayo J.C., Sainz R.M. (2015). Melatonin Uptake through Glucose Transporters: A New Target for Melatonin Inhibition of Cancer. J. Pineal Res..

[259] Iqbal M.A., Bamezai R.N.K. (2012). Resveratrol Inhibits Cancer Cell Metabolism by Down Regulating Pyruvate Kinase M2 via Inhibition of Mammalian Target of Rapamycin. PLoS ONE.

[260] Li W., Ma X., Li N., Liu H., Dong Q., Zhang J., Yang C., Liu Y., Liang Q., Zhang S. (2016). Resveratrol Inhibits Hexokinases II Mediated Glycolysis in Non-Small Cell Lung Cancer via Targeting Akt Signaling Pathway. Exp. Cell Res..

[261] Kueck A., Opipari A.W., Griffith K.A., Tan L., Choi M., Huang J., Wahl H., Liu J.R. (2007). Resveratrol Inhibits Glucose Metabolism in Human Ovarian Cancer Cells. Gynecol. Oncol..

[262] Liu Z., Peng Q., Li Y., Gao Y. (2018). Resveratrol Enhances Cisplatin-Induced Apoptosis in Human Hepatoma Cells via Glutamine Metabolism Inhibition. BMB Rep..

[263] Moreira L., Araújo I., Costa T., Correia-Branco A., Faria A., Martel F., Keating E. (2013). Quercetin and Epigallocatechin Gallate Inhibit Glucose Uptake and Metabolism by Breast Cancer Cells by an Estrogen Receptor-Independent Mechanism. Exp. Cell Res..

[264] Karpova N., Fefilova E., Daks A., Parfenyev S., Nazarov A., Barlev N.A., Shuvalov O. (2025). Dietary Polyphenol Combinations Have a Multifaceted Inhibitory Effect on Metabolic Rewiring and Signaling Pathways in Neuroblastoma. Pharmaceuticals.

[265] Bruntz R.C., Belshoff A.C., Zhang Y., Macedo J.K.A., Higashi R.M., Lane A.N., Fan T.W.-M. (2019). Inhibition of Anaplerotic Glutaminolysis Underlies Selenite Toxicity in Human Lung Cancer. Proteomics.

[266] Zhao J., Zhou R., Hui K., Yang Y., Zhang Q., Ci Y., Shi L., Xu C., Huang F., Hu Y. (2017). Selenite Inhibits Glutamine Metabolism and Induces Apoptosis by Regulating GLS1 Protein Degradation via APC/C-CDH1 Pathway in Colorectal Cancer Cells. Oncotarget.

[267] Abu El Maaty M.A., Dabiri Y., Almouhanna F., Blagojevic B., Theobald J., Büttner M., Wölfl S. (2018). Activation of Pro-Survival Metabolic Networks by 1,25(OH)2D3 Does Not Hamper the Sensitivity of Breast Cancer Cells to Chemotherapeutics. Cancer Metab..

[268] Santos J.M., Khan Z.S., Munir M.T., Tarafdar K., Rahman S.M., Hussain F. (2018). Vitamin D3 Decreases Glycolysis and Invasiveness, and Increases Cellular Stiffness in Breast Cancer Cells. J. Nutr. Biochem..

[269] Zhou X., Zheng W., Nagana Gowda G.A., Raftery D., Donkin S.S., Bequette B., Teegarden D. (2016). 1,25-Dihydroxyvitamin D Inhibits Glutamine Metabolism in Harvey-Ras Transformed MCF10A Human Breast Epithelial Cell. J. Steroid Biochem. Mol. Biol..

[270] Parajuli P., Tiwari R.V., Sylvester P.W. (2015). Anticancer Effects of γ-Tocotrienol Are Associated with a Suppression in Aerobic Glycolysis. Biol. Pharm. Bull..

[271] Stine Z.E., Schug Z.T., Salvino J.M., Dang C.V. (2022). Targeting Cancer Metabolism in the Era of Precision Oncology. Nat. Rev. Drug Discov..

[272] Tomin T., Honeder S.E., Liesinger L., Gremel D., Retzl B., Lindenmann J., Brcic L., Schittmayer M., Birner-Gruenberger R. (2025). Increased Antioxidative Defense and Reduced Advanced Glycation End-Product Formation by Metabolic Adaptation in Non-Small-Cell-Lung-Cancer Patients. Nat. Commun..

[273] Koppula P., Zhuang L., Gan B. (2021). Cystine Transporter SLC7A11/xCT in Cancer: Ferroptosis, Nutrient Dependency, and Cancer Therapy. Protein Cell.

[274] Wu X., Yang M., Zhang H., Yang L., He Y., Cheng X., Zhu G. (2025). Advances in Understanding Renin–Angiotensin System-Mediated Anti-Tumor Activity of Natural Polyphenols. Biomolecules.

[275] Momal U., Shahbaz M., Perween A., Hassan M.H.U., Naeem H., Shahid Z., Hussain M., Imran M., Alsagaby S.A., Al Abdulmonem W. (2026). Anticancer Molecular Mechanisms of Curcuminoids: An Updated Review of Clinical Trials. Food Sci. Nutr..

[276] Almatroodi S.A., Alsahli M.A., Rahmani A.H. (2022). Berberine: An Important Emphasis on Its Anticancer Effects through Modulation of Various Cell Signaling Pathways. Molecules.

[277] Cao Y., Zhang H., Chen X., Li C., Chen J. (2025). Melatonin: A Natural Guardian in Cancer Treatment. Front. Pharmacol..

[278] Ang A., Pullar J.M., Currie M.J., Vissers M.C.M. (2018). Vitamin C and Immune Cell Function in Inflammation and Cancer. Biochem. Soc. Trans..

[279] Hazan S., Dave S., Papoutsis A.J., Deshpande N., Howell M.C., Martin L.M. (2025). Vitamin C Improves Gut *Bifidobacteria* in Humans. Future Microbiol..

[280] Lv H., Wang C., Fang T., Li T., Lv G., Han Q., Yang W., Wang H. (2018). Vitamin C Preferentially Kills Cancer Stem Cells in Hepatocellular Carcinoma via SVCT-2. npj Precis. Oncol..

[281] Zitka O., Skalickova S., Gumulec J., Masarik M., Adam V., Hubalek J., Trnkova L., Kruseova J., Eckschlager T., Kizek R. (2012). Redox Status Expressed as GSH:GSSG Ratio as a Marker for Oxidative Stress in Paediatric Tumour Patients. Oncol. Lett..

[282] Gonenc A., Ozkan Y., Torun M., Simsek B. (2001). Plasma Malondialdehyde (MDA) Levels in Breast and Lung Cancer Patients. J. Clin. Pharm. Ther..

[283] Valavanidis A., Vlachogianni T., Fiotakis C. (2009). 8-Hydroxy-2′ -Deoxyguanosine (8-OHdG): A Critical Biomarker of Oxidative Stress and Carcinogenesis. J. Environ. Sci. Health Part. C.

[284] Chang C., Worley B.L., Phaëton R., Hempel N. (2020). Extracellular Glutathione Peroxidase GPx3 and Its Role in Cancer. Cancers.

[285] DeBlasi J.M., Falzone A., Caldwell S., Prieto-Farigua N., Prigge J.R., Schmidt E.E., Chio I.I.C., Karreth F.A., DeNicola G.M. (2023). Distinct Nrf2 Signaling Thresholds Mediate Lung Tumor Initiation and Progression. Cancer Res..

[286] DeNicola G.M., Karreth F.A., Humpton T.J., Gopinathan A., Wei C., Frese K., Mangal D., Yu K.H., Yeo C.J., Calhoun E.S. (2011). Oncogene-Induced Nrf2 Transcription Promotes ROS Detoxification and Tumorigenesis. Nature.

